# Protocol for a Trial Assessing the Impacts of School-Based WaSH Interventions on Children’s Health Literacy, Handwashing, and Nutrition Status in Low- and Middle-Income Countries

**DOI:** 10.3390/ijerph18010226

**Published:** 2020-12-30

**Authors:** Stephanie O. Sangalang, Shelley Anne J. Medina, Zheina J. Ottong, Allen Lemuel G. Lemence, Donrey Totanes, John Cedrick Valencia, Patricia Andrea A. Singson, Mikaela Olaguera, Nelissa O. Prado, Roezel Mari Z. Ocaña, Rovin James F. Canja, Alfem John T. Benolirao, Shyrill Mae F. Mariano, Jergil Gyle Gavieres, Clarisse P. Aquino, Edison C. Latag, Maria Vianca Jasmin C. Anglo, Christian Borgemeister, Thomas Kistemann

**Affiliations:** 1 Center for Development Research, University of Bonn, 53113 Bonn, Germany; cb@uni-bonn.de (C.B.); thom-as.kistemann@ukb.uni-bonn.de (T.K.); 2 College of Social Work and Community Development, University of the Philippines Diliman, Quezon City 1101, Philippines; shelleyannemedina@gmail.com; 3 Marine Science Institute, University of the Philippines Diliman, Quezon City 1101, Philippines; zjot-tong@gm.gist.ac.kr (Z.J.O.); smariano@msi.upd.edu.ph (S.M.F.M.); 4 School of Earth Sciences and Environmental Engineering, Gwangju Institute of Science and Technology, Gwangju 61005, Korea; 5 National Graduate School of Engineering, College of Engineering, University of the Philippines Diliman, Quezon City 1101, Philippines; aglemence1@up.edu.ph; 6 College of Mass Communication, University of the Philippines Diliman, Quezon City 1101, Philippines; dttotanes1@up.edu.ph (D.T.); mikaelaolaguera@gmail.com (M.O.); 7 National College of Public Administration and Governance, University of the Philippines Diliman, Quezon City 1101, Philippines; cedrickvalencia@yahoo.com (J.C.V.); rfcanja@gmail.com (R.J.F.C.); 8 School of Social Sciences, Ateneo de Manila University, Quezon City 1800, Philippines; patriciasingson97@gmail.com; 9 Kashiwa Campus, University of Tokyo, Chiba 277-0882, Japan; nelissaprado@gmail.com; 10 School of Medicine, Far Eastern University, Nicanor Reyes Medical Foundation, Quezon City 1118, Philippines; rzoca-na@up.edu.ph; 11 College of Business Administration, Adamson University, Manila 1000, Philippines; alfem.john.benolirao@adamson.edu.ph; 12 Department of Sociology, College of Social Sciences and Philosophy, University of the Philippines Dili-man, Quezon City 1101, Philippines; jergilgavieres@gmail.com (J.G.G.); clangaquino3@gmail.com (C.P.A.); 13 College of Engineering, Technological University of the Philippines, Manila 1008, Philippines; latagec@national-u.edu.ph; 14 Department of Psychology, College of Social Sciences and Philosophy, University of the Philippines Diliman, Quezon City 1101, Philippines; mcanglo@up.edu.ph; 15 Institute of Hygiene and Public Health, University of Bonn, 53127 Bonn, Germany

**Keywords:** children’s health, health literacy, malnutrition, water, sanitation, and hygiene

## Abstract

Diarrhea, soil-transmitted helminth infection and malnutrition are leading causes of child mortality in low- and middle-income countries (LMICs). To reduce the prevalence of these diseases, effective interventions for adequate water, sanitation, and hygiene (WaSH) should be implemented. This paper describes the design of a cluster-randomized controlled trial that will compare the efficacy of four school-based WaSH interventions for improving children’s health literacy, handwashing, and nutrition. Interventions consisted of (1) WaSH policy reinforcement; (2) low-, medium-, or high-volume health education; (3) hygiene supplies; and (4) WaSH facilities (e.g., toilets, urinals, handwashing basins) improvements. We randomly allocated school clusters from the intervention arm to one of four groups to compare with schools from the control arm. Primary outcomes were: children’s health literacy, physical growth, nutrition status, and handwashing prevalence. Secondary outcomes were: children’s self-reported health status and history of extreme hunger, satisfaction with WaSH facilities, and school restrooms’ WaSH adequacy. We will measure differences in pre- and post-intervention outcomes and compare these differences between control and intervention arms. This research protocol can be a blueprint for future school-based WaSH intervention studies to be conducted in LMICs. Study protocols were approved by the ethics committees of the University of Bonn, Germany, and the University of the Philippines Manila. This trial was retroactively registered, ID number: DRKS00021623.

## 1. Introduction

### 1.1. Background

Malnutrition, characterized by an imbalance in energy and/or nutrient intake [1], affects ~1 out of 3 people globally [2]. In 2017, 22.2% (150.8 million) of all children <5 years old were stunted [3]. In the same year, while 5.6% (38.3 million) children were overweight, 7.5% (50.5 million) were wasted [3], i.e., acutely undernourished or having low weight-for-height < –2 standard deviation (SD) of the World Health Organization (WHO) Child Growth Standards median [4]. Undernutrition, which is highly prevalent in low- and middle-income countries (LMICs), caused 45% (>29,000) of all deaths in children <5 [5,6]. Overweight and obesity caused ~7% (4 million) of all deaths and 120 million healthy years of life lost (disability-adjusted life years [DALYs]) [6]. Malnutrition, in all its forms, could cost society 5% (US $3.5 trillion) of the global gross domestic product (GDP) [7]. High economic losses disproportionately affect LMICs. Child undernutrition alone cost US $7 million and US $254 million in Swaziland and Uganda, respectively [8].

Preventing children’s malnutrition involves preventing infectious diseases that precipitate imbalanced energy and/or nutrient intake. Diarrhea and soil-transmitted helminth (STH) infections, which affect millions of children in LMICs [9,10], can be prevented by interrupting routes of fecal-oral disease transmission through practicing proper hygiene, especially handwashing, handling food and disposing waste safely, and providing access to clean water for drinking and washing. Interventions for improving water, sanitation, and hygiene (WaSH) have been associated with decreased risk of diarrhea and STH infections, and consequently, decreased risk of malnutrition in various community settings [11,12]. For example, a cluster-randomized controlled trial (cluster-RCT) conducted in rural India found that the construction and promotion of latrines was associated with decreased diarrhea prevalence and increased weight-for-age z-score in children [11]. In Bangladesh, a cluster-RCT showed that an intervention incorporating water quality, sanitation, handwashing, and nutrition was associated with decreased diarrhea and increased height in children [12]. In 2016, 6000 deaths due to malnutrition could have been prevented by improving WaSH [13]. However, it is unclear how WaSH interventions in schools benefit children in LMICs, specifically in megacities, i.e., cities with >10 million inhabitants [14].

The Philippines, an archipelagic country in Southeast Asia, had a population of ~106.7 million in 2018 [15]. The Philippines’ National Capital Region, known as Metro Manila, is identified as a megacity although it is not a single city but a metropolitan area comprising 16 cities. Metro Manila had ~12.9 million inhabitants in 2015 [16], representing 12.1% of the country’s total population. In 2015 Metro Manila’s population density was 20,785 persons per km^2^ [17], or more than 4 times the population density of Beijing in 2014 [18]. Compared to other cities in LMICs, Metro Manila has a unique risk profile as a megacity that is exposed to ≥3 types of natural disasters [14], e.g., typhoons, floods, and volcanic eruptions. Thus, Metro Manila represents an important intersection of human health and the environment. Gaps in environmental health management have contributed to the increased prevalence of environment-related infectious diseases such as diarrhea and STH infections, making Metro Manila a precarious area to live in. This is an important issue because of long-term health consequences, e.g., growth stunting [10]. In 2018 more than half (53.9%) of households in the Philippines was food insecure [19]. In the Philippines the average daily intakes of whole grains and milk (45.5 g/day and 3.7 g/day, respectively) are lower compared to those in Indonesia (51 g/day and 7 g/day, respectively) [3]. Prevalence rates of school-age children’s (6–10 years old) stunting, underweight, wasting/thinness, and overweight-for-height were: 24.5%, 25%, 7.6%, and 11.7%, respectively [20]. In the Asia region, the average stunting rate for children <5 years old is 21.8% but in the Philippines it is >30% [3]. The prevalence of low birth weight is higher in the Philippines (20%) compared to Ghana (14.2%) [3]. The prevalence of wasting for children <5 years old in the Philippines (5.6%) is higher than in Malawi (1.3%) [3]. While undernutrition alone is estimated to cost the Philippines USD $4.5 billion annually [21], the overall cost of hunger was USD $6.5 billion in 2013 [22].

The Philippines Department of Education (DepEd) oversees 54,602 public schools nationwide, hosting >22.6 million children during the School Year 2018–2019 [23]. While public schools receive government funding, they suffer from a lack of teachers and classrooms, and therefore, are overcrowded. The student-to-classroom ratio in Metro Manila public schools ranges from 50:1 to >100:1 [23,24]. This shortage of classrooms has caused the implementation of “double-shift” school-days, wherein one-half of students attend school during a morning shift (6:00 a.m.–12:00 p.m.) and another half attend school during an afternoon shift (12:00 p.m.–6:00 p.m.). The country’s poorest children attend public schools. They are vulnerable to contracting diarrhea and STH infections because they are usually malnourished due to their low socioeconomic status (SES). The school environment, which they are regularly exposed to for prolonged periods, possibly plays a major role in this. Policies have been implemented to achieve adequate WaSH in schools (Box S1), yet there remain sizable gaps in WaSH management--gaps, which threaten the status of schools as health- and education-promoting environments and increase children’s risk of disease.

### 1.2. Objectives

**Figure 1:**
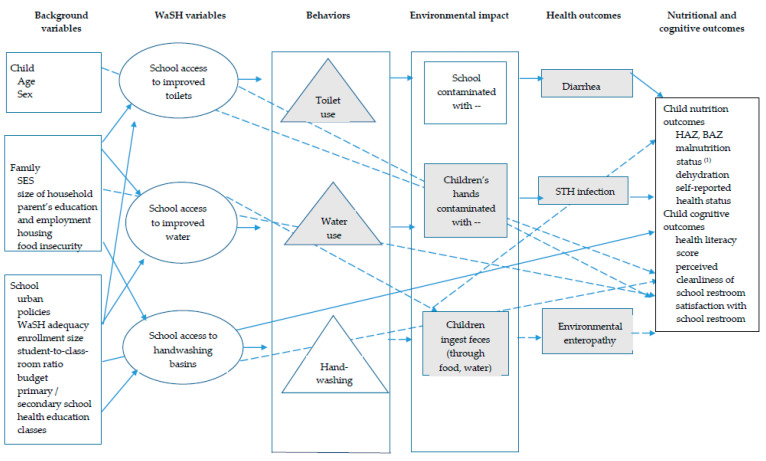
Conceptual framework for water, sanitation, and hygiene (WaSH) and child nutrition, hydration, and cognitive outcomes. Adapted from Dearden et al. [26]. Note: body mass index-for-age z-score (BAZ), height-for-age z-score (HAZ), socioeconomic status (SES); soil-transmitted helminth (STH), water, sanitation, and hygiene (WaSH). We will analyze relationships symbolized by solid arrows, not dashed arrows. Variables that may not be available for analysis during this phase of the research project are symbolized by gray color. ^(1)^ Malnutrition status: stunted, undernourished, and over-nourished.

**Figure 2:**
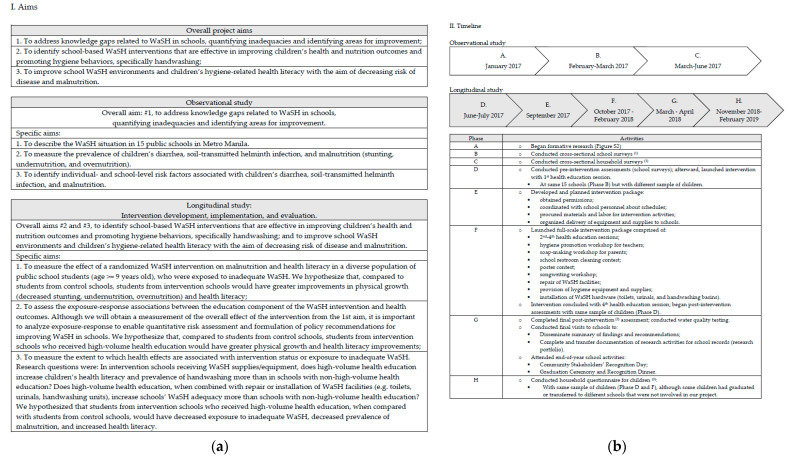
WaSH in Manila Schools project aims and timeline. Note: water, sanitation, and hygiene (WaSH). (**a**) Aims of overall project, observational study, and longitudinal study. (**b**) Timelines for observational study and longitudinal study, including research activities. ^(1)^ Please see References [27]. ^(2)^ Eight months after implementing the intervention, we revisited children from the control and intervention arms for a follow-up assessment that used the same study design as the baseline assessment. We used the same survey instruments to measure: children’s health literacy (Tables S3 and S4) and health and nutrition outcomes; children’s handwashing prevalence; school restrooms’ WaSH adequacy; and schools’ WaSH policies. ^(3)^ In November 2018, 17 months after implementing the intervention, we administered a household questionnaire for children (Table S5), some of whom had graduated and/or transferred to different schools. We completed the household questionnaire in February 2019.

The WaSH in Manila Schools Trial aimed to identify an intervention that could better improve children’s health. Our conceptual framework (Figure 1) depicts our pre-specified hypotheses (Figure 2). To test our hypotheses, we will compare a comprehensive school-based WaSH intervention with no intervention, rather the “standard of care”. This comparison will enable us to estimate a potentially achievable reduction of inadequate WaSH, as well as estimate impacts on malnutrition reduction. Through our description of exposure-response relationships in Metro Manila schools, we will be able to provide estimates of the range of improvements that can be achieved in real-world, LMIC conditions where elements of adequate school WaSH, including improved sanitation, are typically combined with inadequate school WaSH and unimproved sanitation. Results of the trial will be reported in a forthcoming paper (unpublished; manuscript in preparation) [25]. In this paper, we present and discuss the rationale and design of a cluster-randomized controlled trial (RCT) that tested the efficacy of a school-based WaSH intervention. The research protocol we describe in this paper was designed and implemented in the Philippines but it can serve as a blueprint for conducting future intervention studies in other LMICs.

## 2. Research Methodology

### 2.1. Study Overview and Design

WaSH in Manila Schools was a research project aimed at improving children’s environmental health by addressing inadequate school WaSH and promoting health literacy and hygiene practices (Figure 2). We implemented the project from January 2017 to February 2019, first conducting an observational study [27] to assess the WaSH situation in 15 public schools in Metro Manila, and then conducting a longitudinal study to develop, implement, and evaluate WaSH interventions in the same public schools (Figure 2). We conducted a cluster-RCT to address three specific aims (Figure 2) and compare the efficacy of four school-based WaSH intervention packages. Each intervention package consisted of different combinations of four elements: (1) WaSH policy reinforcement, (2) health education, (3) hygiene supplies, and (4) WaSH facilities (e.g., toilets, urinals, handwashing basins) improvements (Table S1).

This was a two-arm cluster-RCT (Figure S1), with one intervention arm and one control arm. The control arm received no intervention, rather the “standard of care”, which we defined as WaSH policy reinforcement, a hygiene workshop for teachers, and two health education sessions. The intervention arm was further divided into four groups (A, B, C, and D), based on the volume (low, medium, or high) of health education provided. Cluster units were public schools located in three cities of Metro Manila, Philippines, where many people, including a majority of public school children, were exposed to inadequate WaSH.

### 2.2. Study Population

For primary schools, we recruited students in grades 5 and 6 and for secondary schools in grades 7 and 10. These grade levels were chosen because we wanted to involve students who were: (1) developmentally mature enough to use and have perceptions about school WaSH facilities; (2) capable of responding independently or with minimal assistance to our questionnaire using a tablet or smartphone; and (3) able to actively participate in intervention activities (for inclusion and exclusion criteria for students, see Table S2).

### 2.3. Sampling and Recruitment

**Figure 3:**
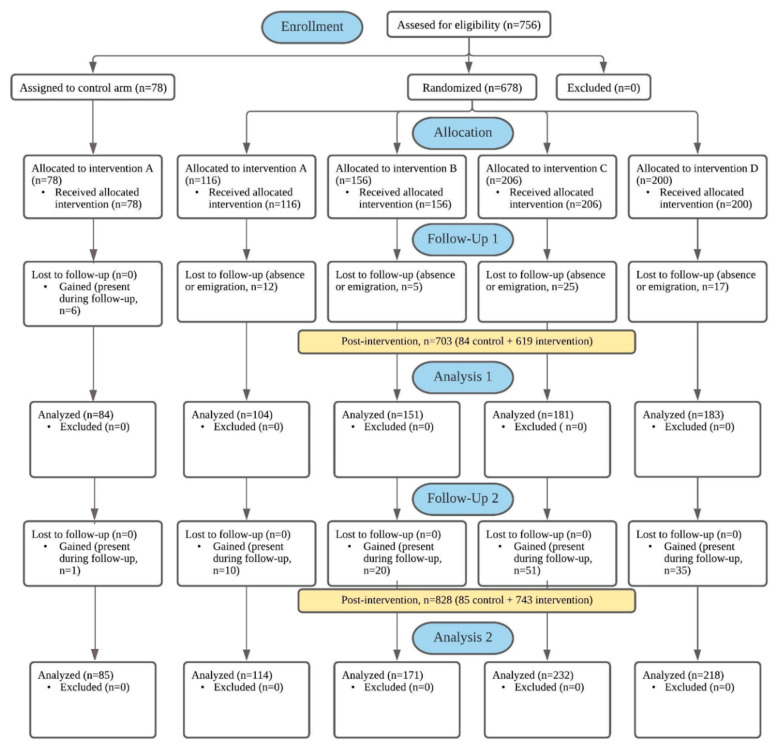
Assignment of participants and completed follow-up: flow diagram based on CONsolidated Standards of Reporting Trials (CONSORT) [28].

We conducted formative research (Figure S2) to prepare for the implementation of our intervention. We previously described how we estimated the sample size using the Lynch formula [27]. The target population was all the public school children in Metro Manila, where in School Year 2014–2015 a total of 2,059,447 public school children (1,373,852 elementary and 685,595 secondary school children) were enrolled [23]. We inflated the sample by 30% and 45% to account for nonresponse and refusal, respectively, and then inflated the sample by another 5% to account for differences in schools’ enrollment sizes. The target sample size was N = 760; we enrolled 756 students at baseline and 703 students at the 8-month follow-up (retention rate: 86%) (Figure 3).

**Figure 4:**
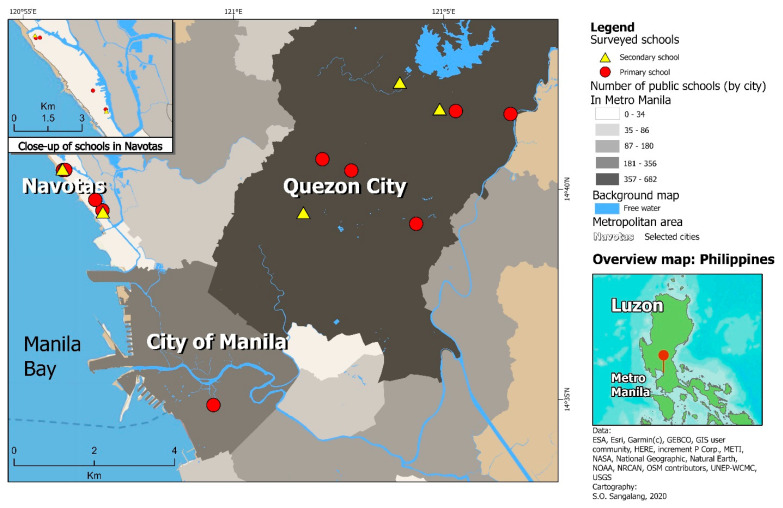
Map of study area with points marking the location of study schools. The map shows the study area in the National Capital Region, also known as Metro Manila, in northern Philippines, with study schools plotted as points. The red pushpin marker in the lower left inset map indicates where the location of the study area is within the Philippines, specifically in the northern most island group of Luzon.

We selected schools located in Manila, Navotas, and Quezon City (Figure 4) because they were geographically and demographically representative of cities in Metro Manila. We recruited the school principals from the 15 public schools that participated in our previous observational study [27]. We used convenience, rather than probabilistic, sampling, choosing to revisit the same 15 schools because of existing relationships of trust and cooperation with school principals and personnel. These factors can facilitate communication and collaboration, which were crucial because we aimed to maintain a long-term working relationship with schools in spite of our limited resources in time, personnel, and materials. We previously described our multi-stage cluster sampling strategy [27]. Based on the school’s enrollment size, 1–2 class sections were selected to obtain a target sample of ~50 students per school. We recruited entire class sections as a whole rather than groups of students from multiple class sections so as not to interrupt on-going classroom instruction with our research activities. We decided not to re-recruit the students from our previous observational study as a new school year had begun, resulting in students advancing up to the next grade level (e.g., from grade 5 to 6) and moving to different class sections, as well as some students transferring to different schools that were not involved in our study. No monetary reimbursement was offered to school principals, and all schools were compensated (Table S6) with printed research portfolios, certificates of appreciation, classroom viewings of an educational film promoting handwashing, and hand hygiene gel.

### 2.4. Ethics Approval

The ethics committees of the University of Bonn, Germany (Number 216/16), and the University of the Philippines Manila (Number 2017–0113) provided written approvals for the study. Before conducting the school surveys we obtained written approval from the DepEd through division superintendents. Written informed consent was provided by school principals in loco parentis, i.e., in the place of a parent, for the children’s participation. Prior to conducting our study, we described to the children the study procedures in an easy to understand way using the local language (Filipino/Tagalog). We emphasized that participation in our study was voluntary and that anyone could decide to stop participating in the study anytime. We encouraged children to ask the research team any questions they might have. We accepted children’s affirmative agreement (“assent”) to participate in the study as soon as they actively showed a willingness to join the research project by completing the questionnaire and undergoing a health examination. We retroactively registered our study in the German Clinical Trials Registry (DRKS), ID number: DRKS00021623. Study registration was delayed due to logistical and human resource constraints. We strictly followed the protocols, which were developed before data collection. This ensured that the study’s quality was not impaired.

### 2.5. Data Collection

#### 2.5.1. Assessing Primary Intervention Outcomes

First we assessed children’s health literacy, measured by two continuous variables (hygiene literacy and handwashing literacy scores) via questionnaire (Tables S3 and S4). Second we assessed child growth, measured by continuous variables (z-scores for height-for-age [HAZ] and body mass index-for-age [BAZ]) and binary variables (prevalence of malnutrition [stunting, undernutrition, and overnutrition]), via anthropometry (Table 1). HAZ is a linear growth indicator that indicates if the child has the appropriate stature for his/her age, while BAZ is an indicator of growth that indicates if the child has the appropriate ratio of weight and height for his/her age, according to WHO guidelines [4,29,30,31,32]. We used the WHO AnthroPlus (for children 5–19 years old) software (version 3.2.2., WHO, Geneva, Switzerland) to obtain HAZ and BAZ for each child. Third we assessed handwashing prevalence via observation according to standard operating procedure (SOPs) (Figure S3).

#### 2.5.2. Assessing Secondary Intervention Outcomes

First we assessed children’s overall health status, extreme hunger prevalence, and satisfaction with and perceived cleanliness of schools’ WaSH facilities, measured by binary variables, via questionnaire (Table S6). We assessed acute dehydration, measured by incidence of highly concentrated urine (U_sg_ ≥ 1.020) [33,37,38] (Table 1), via urinalysis using urine test strips (Insight Urinalysis Reagent Strips, Acon Laboratories Inc., San Diego, CA, USA). Urine test strips are an affordable, easy to use, and reliable way to perform urinalysis in field settings [39]. Second we assessed school principals’ satisfaction with schools’ WaSH facilities and children’s hygiene practices, measured by binary variables, via interview. Third we assessed, via observation, schools’ WaSH adequacy, measured by continuous and binary variables describing availability, accessibility, cleanliness, and functionality, according to guidelines from the Philippines DepED and Department of Health (DOH) [35,40]. We previously reported the data collection tools we used for conducting health examinations, interviews with school principals, and school restroom inspections [27]. Data collection tools were pilot-tested and improved prior to beginning this trial.

During our previous observational study, we found that children were avoiding or not using school restrooms, possibly due to a perceived lack of cleanliness or lack of privacy. Findings indicated that avoidance and non-use of school restrooms increased children’s risk of diarrhea. Thus, we aimed to see if our intervention was effective in improving children’s perceptions about school restrooms, with the hope that children would use school restrooms rather than avoid using them. Also, we found that school WaSH policies were in place, yet school principals reported being unsatisfied with WaSH facilities and children’s hygiene behaviors. School principals’ perceptions about WaSH could affect efforts to implement, maintain, and sustain improvements, all of which influence children’s health and hygiene behaviors. Thus, we aimed to see if our intervention was effective in improving school principals’ perceptions about school WaSH facilities.

#### 2.5.3. Baseline Survey and Randomization

At baseline, we conducted a four-part school-based survey (Figure S3) to measure: (1) children’s health literacy and nutrition status; (2) handwashing prevalence; (3) school restrooms’ WaSH adequacy; and (4) schools’ WaSH policies. We developed a self-administered health literacy questionnaire for children in English and then translated it to Tagalog (Filipino language) (Tables S3 and S4). We made one version for primary and one version for secondary school children. The QuickTapSurvey^©^ app (Formstack LLC, Fishers, IN, USA), installed on password-protected tablets and smartphones, was used to administer all survey instruments. It is a cloud-based data collection tool that allows for simultaneous offline data entry and stamping with global positioning system (GPS) coordinates, date, and time. We applied validation rules and range limits for precise data entry.

The research supervisor conducted a full-day (8 h) initial training workshop (Table S7) for research assistants before conducting school surveys. Each school survey was conducted by a team of 5–7 rotating research assistants, directly managed by the research supervisor. To begin the school survey, we first explained the study’s aims, objectives, and methods to the children in Tagalog (Figure S3). We emphasized that participation in our study was voluntary and anonymous, and that results would remain confidential and have no impact on anyone’s school grades. Children’s full name, date of birth, and telephone number were collected and stored in a separate Microsoft© Excel spreadsheet secured in password-protected tablets. Next, we assigned children unique identification (ID) numbers and gave them each three tickets that were printed with their ID number. The ticketing system was a quality control measure that ensured that all children provided all the requested data. Children were instructed to give one ticket to the assigned research assistant at every station that they visited during the school survey (Figure S3).

After obtaining data from the children, research assistants completed the school restroom inspection checklist, took photos of WaSH facilities, and interviewed school principals or representatives, inputting all data into the app. We obtained school administrative data, e.g., budget for maintenance and other operating expenses, from the DepEd. At the end of each workday, research assistants discussed their work with each other, shared lessons learned, and identified areas for improvement. Upon accessing an internet hotspot, we used the app to transmit anonymized data to a central database via cloud storage. Research assistants periodically checked uploaded data to verify the completeness of data upload and identify discrepant, missing, or duplicate data. When needed, appropriate remedial action was taken, e.g., re-training research assistants or clarifying instructions for children.

After completing baseline surveys, we assigned two schools to the control arm and 13 schools to the intervention arm (Figure 3). We purposefully assigned two schools to the control arm because one school had a school principal who directly asked us to participate in our previous observational study. The other school was integrated (offering all possible grade levels, from kindergarten to grade 12) and was the location where we had pilot-tested our survey instruments before the observational study. We randomly allocated schools in the intervention arm to one of four groups. One research assistant and the research supervisor conducted randomization in Microsoft^©^ Excel as follows: (1) In one column, we listed the names of the 13 schools in the intervention arm, with one row representing one school. (2) In another column, we listed the four intervention groups (A, B, C, and D), with one row representing one group. We previously determined how many schools would be allocated to each group (Table S1). (3) In a third column, we used Excel’s random number function and ranked the schools, assigning each school to either group A, B, C, or D (Table S1). Except for the research supervisor, all research assistants, study participants (children), school personnel, and parents were blinded to the assignment of schools to the intervention arm and subsequent random allocation to intervention groups A–D.

#### 2.5.4. Follow-Up and Other Assessments

Eight months later, we conducted a follow-up assessment by implementing the same methodology (i.e., 4-part school-based survey) that we used at baseline (Figure S3) and measuring the same types of outcomes (e.g., children’s health literacy and nutrition status, handwashing prevalence, school restrooms’ WaSH adequacy, and schools’ WaSH policies). Nine months later (17 months after the first health education session), we conducted a household questionnaire for children (Table S5). Thus, we were able to assess risk factors at the individual-, school-, and home-levels (Table 2).

We expected the intervention to influence children’s knowledge, behavior, and health outcomes. We defined indicators of the intervention’s impact as changes in children’s primary and secondary outcomes. We measured the difference between outcomes from the eight-month follow-up and baseline. We compared differences between control and intervention arms. We collected samples for water quality testing from study schools and a separate sample of households (located in the school neighborhood) after post-intervention assessments, as previously reported [27].

### 2.6. Intervention

The four parts of the intervention were: (1) WaSH policy reinforcement, (2) health education, (3) hygiene supplies, and (4) WaSH facilities improvements (Table S1). We implemented the intervention over eight months, from June 2017 to February 2018 (Figure 2). The development of the intervention was informed by findings from our previous observational study and baseline survey, as well as inputs from research assistants with expertise in the local context and stakeholders, e.g., school principals, teachers, and janitors. We also conducted opinion polls with students. It was important for us to use participatory research methods that engaged stakeholders so that we could assess whether our intervention would be acceptable or sustainable after the research project ended.

School principals were presented with a research portfolio containing recommendations for enforcing existing WaSH policies (Figure S4). We conducted one-hour hygiene promotion workshops for teachers, explaining the importance of improving WaSH in schools to achieve the United Nations’ (UN) Sustainable Development Goal (SDG) 6 [41], related to WaSH, and complying with the DepEd’s Order No. 10 [40], related to WaSH in schools. The workshop was valuable for teachers because it helped them gain new ideas about how to present to children specific health education topics, and see how big of a role classroom activities play in achieving schools’ WaSH policy goals.

At the core of our intervention was health education. Research assistants conducted interactive verbal presentations for children, who, depending on which intervention group they belonged to, received two to four health education sessions that lasted one hour each. The content of the health education sessions (Figure S5) was based on the existing DepEd curriculum and open educational resources from, e.g., the United States Centers for Disease Control and Prevention (CDC) and Environmental Protection Agency (EPA). Microsoft^©^ PowerPoint slides, with visually appealing graphics to attract children’s attention, were created (Figure S5). Each health education session included a brief lecture, class discussion, and a trivia game. We also incorporated role-playing, other games, and singing the “Happy Birthday” song to teach children the correct duration of time for handwashing. Besides health education sessions, we used mixed methods to pique children’s interest, invite active participation, and encourage a sense of empowerment about hygiene at the personal- and environment-levels. For example, we implemented poster-making and school restroom-cleaning contests, as well as songwriting workshops specifically for children and adolescents from secondary schools. Research assistants also developed an original educational video, filmed in Tagalog, called “Hygiene Heroes”, to promote proper handwashing (Film S1). After launching the intervention, we promoted adherence with various communication strategies (Table S8).

### 2.7. Trial and Data Management

Our trial involved a non-invasive, non-pharmacological intervention that posed little risk to participants. Thus, we saw no need for trial steering or data monitoring committees, nor did we see a need for interim data analysis. The research supervisor, who was based at the University of the Philippines Diliman for the entire duration of the trial, led the day-to-day management of data. The research team conducted the trial according to protocol, as approved by ethics committees, to ensure the safety of study participants, maintain the trust of stakeholders, preserve data quality, and maintain the integrity of the project.

Research assistants collected data using password-protected tablets and smartphones offline. We uploaded the data daily to the secure QuickTapSurvey^©^ server, and then refreshed tablets and smartphones, deleting all data from the devices. We downloaded data from the QuickTapSurvey server as Microsoft^©^ Excel files, which eliminated the need for manual data entry and its associated risks for error. We manually inspected data for discrepancies and inconsistencies and addressed these according to the SOPs (Figure S3). We used key matching data (children’s date of birth and telephone number) to link data from children’s questionnaires with data from health examinations.

#### 2.7.1. Protocol Standardization and Data Quality Control

To ensure the standardization of research activities within and between cities, across all data collection periods, as well as within and between various teams of research assistants, we used SOPs and a detailed school visit itinerary (Figure S3). The research supervisor explained and demonstrated the SOPs and itinerary in English and Tagalog during an in-person training workshop with research assistants before conducting field research. The research supervisor verified research assistants’ comprehension by return-demonstration. The research supervisor directly oversaw data collection and provided onsite, real-time feedback, providing additional training as needed. Data quality was monitored regularly, and discrepancies were immediately discussed and resolved within the research team. Research assistants were encouraged to propose solutions and workflow improvements.

#### 2.7.2. Dissemination

We discussed preliminary results with project partners and provided research portfolios, containing a summary of results, policy recommendations, and outline for an action plan, to school principals for dissemination to schoolteachers, parents, and children. The research portfolio was valuable for school principals because it helped them to describe their school’s progress toward WaSH-related goals, set by the DepEd. In the Philippines, we presented our methods and summary of interventions to a non-government organization and an international organization. We presented preliminary results at international conferences. Full results will be reported in a forthcoming paper [25].

### 2.8. Statistical Analysis

Because our study contains multiple arms (1 control and 4 intervention arms), it is necessary to develop a strategy for conducting statistical analysis that takes into account the study’s 5 arms, parallel design, and unequal allocation ratio between control and intervention arms. We will use an adjustment method to control for type I error due to the study’s multiple-arm design. Because we simultaneously assessed multiple outcomes, we will need to adjust for a possible multiple comparisons effect. One possible adjustment method is the Benjamini–Hochberg procedure [42], which controls for the false discovery rate (10%). We used unequal allocation, making the intervention group larger than the control group, because we aimed to increase the precision of the intervention comparison. Also, we aimed to make the study more acceptable to study participants and researchers by decreasing the likelihood of being allocated to the control group and not receiving the intervention. Because we aim to know whether and how interventions differ, we will not use a single global test of significance, which would compare all groups simultaneously. Rather we will use a dose-response model to examine trends. We will conduct all analyses by using the originally assigned intervention status (i.e., intention to treat), comparing each intervention arm against the control arm.

We will examine the role of missing data in the interpretation of findings. If we find no statistically significant difference between children who were missing data and children who were not missing data for key outcomes, then we will conclude that data were missing at random (MAR), though not missing completely at random (MCAR). MAR means that all data, within groups defined by the observed data, had an equal chance of being missing, and that the reason why data were missing is due to a known characteristic of the data themselves [43]. In our study, the possible reasons for MAR will likely be: nonresponse (some children did not answer all of the questions in the questionnaire) or loss to follow-up due to school absence or discontinued school enrollment because of drop-out, graduation, or relocation. On the other hand, MCAR will less likely be found in our study because it means that all data had an equal chance of being missing and that the reason why data were missing is not due to the data themselves [43]. In field research, this may be an unrealistic occurrence. We will consider using multiple imputation (MI) or full information maximum likelihood (FIML) to handle MAR data. An advantage of FIML is that it uses all available data provided by a given study participant. We will use Stata, version 15 (StataCorp., College Station, TX, USA), for all data analyses.

#### 2.8.1. Descriptive Analyses

We will conduct descriptive analyses, pre- and post-intervention, to measure study participation, demographic characteristics, and outcomes of interest. We will report baseline characteristics for all five study arms. For each primary and secondary outcome, we will report results (e.g., means, proportions) for each arm, including the estimated effect size and precision. To describe exposure to inadequate WaSH at school and at home, we will measure frequencies and interquartile ranges (IQRs) relevant to schools’ and homes’ WaSH facilities. Data from school inspections will be summarized at the school-level by measuring the mean scores of individual facility inspections. To describe outcomes of poor health literacy, poor handwashing, and malnutrition, we will measure prevalence rates using contingency tables with estimates of standard error (SE) and precision.

#### 2.8.2. Inferential Statistical Analyses

To address study aims 1 and 3 (Figure 2), we will compare the study arms, using two-sided tests for primary outcomes. For continuous variables, we will use the *t*-test to calculate the mean weight differences between the control and intervention arms. For binary variables, we will use Poisson regression to compare the control and intervention arms according to their relative risk (RR). We will use multiple logistic regression models to analyze exposure-response: g = (E[Ai]) = β0 + β1Bi + γCi, where Ai is the primary outcome of interest, g( )  is the appropriate link function (identity for height and weight, logistic for stunting and poor health literacy), Bi is the continuous exposure of interest, and Ci is the vector of confounders. We describe proposed models in Box S2 and will describe full models in a forthcoming paper [25].

To assess risk factors of health, health literacy, and handwashing outcomes, we will use multiple logistic regression models that account for school-level clustering, estimate precision with 95% confidence intervals (CI), and *p*-values, and produce adjusted odds ratios (aORs). Clustering by school takes into account potential intragroup correlation among children from the same school and enables us to adjust the SE of estimates. aORs control for possible confounders (e.g., children’s age and sex, parent’s educational background and employment status, presence of toilet and handwashing basin inside home, household food insecurity). We will use confounders to calculate an adjusted effect estimate to account for baseline differences between the control and intervention arms. We will use three multiple logistic regression models (Box S2): (1) model A for poor hygiene literacy only, poor handwashing literacy only, and both poor hygiene literacy and poor handwashing literacy; (2) model B for malnutrition, i.e., stunting only, undernutrition only, and overnutrition only; and (3) model C for poor health status only, extreme hunger only, and both poor health status and extreme hunger.

We will use generalized linear mixed models (GLMMs), which are appropriate for longitudinal data, to determine if the intervention was effective in improving our outcomes of interest. GLMMs are useful for measuring intervention impacts in complex datasets, i.e., when different types of variables (e.g., binary, continuous) are assessed at different time periods, particularly when assessments are made at the group- rather than individual-level. We will use GLMMs to estimate the effect of each intervention compared to the control group. To measure the intervention’s impact, we will estimate differences between pre- and post-intervention outcomes.

## 3. Discussion

Inadequate-WaSH-related diseases, specifically malnutrition, diarrhea, and STH infection, continue to be leading causes of child mortality and morbidity, particularly in LMICs [5,10,13]. One reason may be that many knowledge gaps still exist and hinder progress on school WaSH. For example, evidence about the relationship between health literacy and handwashing has been limited to adults [44] and older adolescents (age 16–19 years old) [45]. Little is known about how school WaSH impacts child growth and what kind of framework is needed to guide the development of child malnutrition prevention programs. Evidence is limited on which combinations of WaSH interventions could be implemented to achieve optimal health for urban poor children.

We have performed one of the most comprehensive assessments of school WaSH, children’s WaSH-related knowledge, attitudes, and practices (KAP), malnutrition, and risk factors at the individual-, school-, and home-levels. We will fill data gaps on school WaSH facilities, quantifying available resources and deficiencies and identifying areas for improvement. Data from our monitoring of school WaSH facilities will further the understanding contributed by previous studies that were limited by cross-sectional study designs [46,47]. To our knowledge, our study was the first school-based WaSH intervention trial to be conducted in multiple cities of Metro Manila using a common protocol, which will enable the comparability, transferability, and generalizability of the findings to similar LMIC settings. We will be among the first to report the impacts of school WaSH on malnutrition in an under-assessed population of children and younger adolescents (13–15 years old) from a tropical megacity in a LMIC. Our study will show how children’s physical growth could be improved by school-based WaSH interventions, providing key information for malnutrition prevention programs. This is crucial for populations in LMICs, where the prevalence of non-communicable diseases (NCDs), such as malnutrition, is rising rapidly and steeply [2]. By including indicators of malnutrition, food and water insecurity, and policy effectiveness, our study will highlight the need for a new framework to assess the long-term health impacts of inadequate school WaSH. By identifying risk factors of poor health literacy, our study will also address an urgent need to create evidence-based strategies aimed at promoting behavior change, i.e., hygiene practices, that can last beyond childhood and endure along the life course into adulthood.

Our study included intervention components (e.g., health education, poster-making and restroom cleaning contests, promotion of hygiene practices through behavioral reinforcement) that are likely to be operationalized at scale in similar high-risk, low-resource settings in LMICs because they were feasible, acceptable, and affordable. Our study will support using best practices in public health promotion and enhancing local research capacity. We provided local researchers with training about: community health, population-based research, and participatory research methods; assessments of child growth and cognitive development; use of noninvasive markers to evaluate hydration status.

The interpretation of our study findings will likely be limited because of bias due to the non-random recruitment of schools and non-random sampling of children, and the lack of randomization when we assigned school clusters to control or intervention arms. We used outcome measures that could increase the risk of bias by misclassifying children or not controlling for confounding. We used self-reported outcome measures to assess food insecurity and socioeconomic status. We used urine test strips to assess dehydration. However, we did not corroborate or triangulate findings with other sources of data. It is also possible that our assessment of health literacy via questionnaire would introduce bias. We will need to account for many missing responses due to children dropping out of our study because they graduated and/or transferred to a different school. We collected data on many outcomes and will likely run the risk of seeing a “multiple comparisons effect”. This may occur because as the number of outcome measurements increases, so does the tendency of seeing a false-positive [48].

### Implications for Research and Policy

More studies are needed to improve health literacy assessment, expanding its scope to include more relevant content. It would be helpful to corroborate observed handwashing with children’s self-reported handwashing, not just after using the toilet but also before eating. Another option would be to measure a handwashing-proxy, e.g., monitoring the usage of water, soap, and paper/towels in schools as an indicator of children’s handwashing. Enhanced measurement of dehydration ought to be explored by using a refractometer instead of urine test strips, and then corroborating findings with observed clinical signs of dehydration and children’s subjective report of thirst. Sound study designs are needed to improve the generalizability of findings and reduce the risk of bias. It would be helpful to randomly recruit schools and children and randomly assign school clusters to control and intervention arms. It would be useful to design a cohort study that would follow children into adulthood to gain a better understanding of the long-term effects of inadequate WaSH on human growth and nutrition status. Studies are needed to describe the relationship between schools’ menstruation management services and children’s health and education outcomes.

Our findings will demonstrate the need for improved development and enforcement of school WaSH policies, as well as health education and hygiene practices. When synthesized with data from formative research on the barriers and facilitators of handwashing and the use of school WaSH facilities, our findings will enable health practitioners, educators, and other researchers to better promote adherence to proper handwashing. Specifically, our work can inform efforts to improve hygiene education and prevent the neglect of school WaSH facilities by promoting proper operation, maintenance, cleaning, and adequate financing. Our study is very timely and relevant, offering evidence to decision-makers who are responsible for managing the safe reopening and operation of schools during the Corona disease 2019 (COVID-19) pandemic. Our results will provide support for maintaining school WaSH facilities, including water security, and above all, promoting handwashing. Threats to children’s health in relation to school WaSH have recently been exacerbated by the COVID-19 pandemic, especially in resource-constrained settings like public schools in LMICs. Urgent, evidence-based action is required to protect vulnerable populations of children in these settings where citizens face a disproportionately high risk for COVID-19-related death and disease, in addition to severe economic, environmental, and sociopolitical consequences [49,50]. The research protocol described in this paper can serve as a blueprint for future intervention studies to be conducted in other LMICs.

## 4. Conclusions

Effective school-based WaSH interventions are needed to better protect children’s health, especially in LMICs. The design of this cluster-RCT aims to measure the effectiveness of a comprehensive school-based WaSH intervention by measuring impacts on children’s health literacy, handwashing, and nutrition and comparing impacts across study arms that received different volumes (low, medium, or high) of health education. This will enable a deeper understanding about how school WaSH can be better managed to promote children’s health and prevent disease, particularly in precarious environments constrained by limited resources such as urban poor megacities in LMICs.

## Figures and Tables

**Table 1:** Operational definitions.

Indicator		Definition	Reference
Malnutrition			
Stunting	Stunted	HAZ < −2	[4,31,32]
Undernutrition	Underweight (“thin”)	−3 < BAZ < −2	[4,29,30]
	Wasted (“severely thin”)	BAZ < −3	[4,29,30]
Over-nutrition	Overweight	1 < BAZ < 2	[4,29,30]
	Obese	BAZ > 2	[4,29,30]
Acute dehydration Concentrated urine Moderately concentrated urine Severely concentrated urine WaSH adequacy		sg ≥ 1.020 sg = 1.025 sg = 1.030	[33] Category used in present study Category used in present study
Sanitation			
Improved	Facility that hygienically separates human excreta from human contact	e.g., flush toilet, pour-flush latrines, ventilated improved pit latrines and pit latrines with a slab or covered pit	[34]
Unimproved	Facility that does not hygienically separates human excreta from human contact	e.g., pit latrines without slabs or platforms or open pit, hanging latrines, bucket latrines, open defecation, disposal of human feces with other forms of solid waste	[34]
Male student-to-toilet ratio	Less than 50 students	1 toilet, 1 urinal, 1 handwashing basin	[35]
	50 or more students	2 toilets, 1 urinal, 2 handwashing basins	[35]
	For each additional 100 students	1 toilet, 1 urinal, 1 handwashing basin	[35]
Female student-to-toilet ratio	Less than 30 students	1 toilet, 1 handwashing basin	[35]
	30–100 students	2 toilets, 2 handwashing basins	[35]
	For each additional 50 students	1 toilet	[35]
	For each additional 100 students	1 handwashing basin	[35]
Male student-to-toilet ratio	50 students	1 toilet, 1 urinal (or 50 cm of urinal wall)	[36]
Female student-to-toilet ratio	25 students	1 toilet	[36]
Student-to-handwashing basin ratio	N/A	N/A	No WHO guidelines available
Male student-to-toilet ratio	Low	≤ 50:1	Category used in present study
	Medium	51:1–100:1	Category used in present study
	High	≥ 101:1	Category used in present study
Female student-to-toilet ratio	Low	≤ 50:1	Category used in present study
	Medium	51:1–100:1	Category used in present study
	High	≥ 101:1	Category used in present study
Student-to-handwashing basin ratio	Low Medium High	≤ 50:1 51:1-150:1 ≥151:1	Category used in present study Category used in present study Category used in present study

Note: body mass index (BMI); body mass index-for-age Z-score (BAZ); Department of Health Philippines (DOH); height-for-age Z-score (HAZ); not applicable (N/A); urine specific gravity = U_sg_; water, sanitation, and hygiene (WaSH); weight-for-age Z-score (WAZ); World Health Organization (WHO).BAZ is BMI, which is calculated by weight (kg)/[height (m)]^2^. We measured BAZ instead of WAZ because the latter is appropriate only for children < 5. We used BAZ and HAZ measurements, along with children’s age, to calculate anthropometric indices, which were computed as Z-scores (the number of standard deviations [SDs] in relation to the mean of the standard population). When computing Z-scores, we referred to the 2007 WHO database for child growth standards [4,29,30,31,32,33]. We used the WHO AnthroPlus (for children 5–19 years old) software (version 3.2.2., WHO, Geneva, Switzerland) to obtain HAZ and BAZ for each child. We defined acute dehydration as having highly concentrated urine defined as urine sg ≥ 1.020 [33]. We measured urine sg via point-of-care urinalysis using urine test strips as described in Figure S3. We measured urine sg as a continuous variable and as a binary variable: concentrated urine, sg ≥ 1.020 (yes/no), moderately concentrated urine, sg = 1.025, and highly concentrated urine, sg = 1.030). Urine sg estimates the ratio of solutes (e.g., electrolytes, nitrogenous chemicals) compared with distilled water, which has a sg of 1.000. The normal range of urine sg is 1.003–1.030, with higher numbers indicating a greater concentration of solutes and, consequently, decreased hydration (known as “dehydration”) [33]. The gold standard for measuring urine sg is urine osmolality, although this test is invasive, expensive, and not practical in field settings. We defined children as dehydrated if urine sg ≥ 1.020. This cutoff corresponds to a urine osmolality of > 800 mOsm/kg H_2_O, which is the cutoff used in previous studies involving dehydrated children [37,38].

**Table 2:** Data collection during the WaSH in Manila Schools project.

Instrument	Types of Data Collected	Frequency and Time Period (s)
WaSH-related knowledge, attitudes, and practices (KAP) questionnaire for children	Self-reported: Demographics Perceptions about and use of school WaSH facilities Hygiene behavior (e.g., handwashing) Health history Food security	1 time: February–March 2017
Health examination (form) for children	Observed: Height Weight Urine specific gravity	3 times: February–March 2017; different sample of students during June–July 2017 and February–March 2018
Interview (form) for school principal ^(1)^	Self-reported: Perceptions about school WaSH facilities and students’ hygiene WaSH-related policies	2 times: March–June 2017; November–December 2018
School restroom inspection checklist	Observed: Availability, quantity, and quality of school WaSH facilities	2 times: February–March 2017; September–December 2018
Interview (form) for parent/guardian	Self-reported: Demographics Food security Hygiene behavior (e.g., handwashing)	1 time: March–June 2017
Home restroom inspection checklist	Observed: Availability and quality of home WaSH facilities	1 time: March–June 2017
Health literacy questionnaire for children	Self-reported: Demographics Understanding of hygiene concepts and handwashing	2 times: June–July 2017 and February–March 2018
Handwashing observation form	Observed: Children’s handwashing behavior	2 times: October 2017; February–March 2018
Water quality testing form	Observed: Indicators of water quality	1 time: April 2018 ^(2)^
Household questionnaire for children	Self-reported: Demographics Perceptions about school and home WaSH facilities Hygiene behavior (e.g., handwashing) Health and nutrition status Food security Exposure to second-hand smoke Home and household characteristics Parent/guardian’s education and employment	1 time: November 2018–February 2019
N/A	Secondary school administration data ^(3)^	Various

Note: Department of Education Philippines (DepEd); Philippines Department of Health (DOH); knowledge, attitudes, and practices (KAP); maintenance and other operating expenses (MOOE); water, sanitation, and hygiene (WaSH); World Health Organization (WHO).We assessed risk factors for desired outcomes at the individual-, school-, and home-levels. At the individual-level, we assessed demographics, self-reported health status, history of extreme hunger (e.g., feeling too hungry to fall asleep), exposure to secondhand smoke at home, and perceptions of school WaSH facilities. At the school-level, we measured the adequacy of WaSH facilities by counting the number and assessing the quality (e.g., cleanliness, functionality) of toilets, urinals, and handwashing units. We estimated student-to-toilet and student-to-handwashing unit ratios based on the DOH guidelines (Table 1). We decided not to base our estimations on the WHO guidelines (Table 1) because public schools in many parts of the Philippines, similar to other LMICs, currently have limited capacity to effectively address the over-crowding of students on school campus. We asked school principals about WaSH-related school policies, e.g., “Is there a school policy to clean the students’ restrooms daily?” [27]. We retrieved secondary school administrative data from school secretaries and the DepEd. At the household-level, we assessed the family’s demographics (e.g., the number of adults and children in the home), food insecurity, experience of having to ask/beg for food; parent’s employment status, education, literacy, history of physical or intellectual disability; access to and adequacy of the home’s WaSH facilities. To assess the family’s socioeconomic status we examined ownership of household appliances and electronic devices (e.g., refrigerator, mobile phone), ownership of transportation vehicles, most frequently used mode of transportation to school, material of the home’s flooring, and availability of electricity. Eight months after implementing the intervention, we revisited children from the control and intervention arms for a follow-up assessment that used the same study design as the baseline assessment. We used the same survey instruments that we used in the previous observational study [4] to measure: children’s health literacy and nutrition outcomes, handwashing prevalence, school restrooms’ WaSH adequacy, and schools’ WaSH policies. Seventeen months after implementing the intervention, we administered a household questionnaire for children, some of whom had graduated and/or transferred to different schools since participating in the previous follow-up assessment. ^(^^1)^ Some school principals had left their respective schools by the time we conducted our follow-up interviews. Thus, the pre- and post-intervention samples of school principals were not comprised of the exact same individuals for all schools. ^(2)^ In our analyses, we included secondary water quality testing data that was collected in 2016 for one school from the control arm. ^(3)^ School administration data: e.g., enrollment, number of classrooms, and MOOE budget.

## Data Availability

Not applicable.

## Supplementary Materials

The following are available online at https://www.mdpi.com/1660-4601/18/1/226/s1: Box S1: Policies relevant to WaSH in schools, Box S2: Equation and proposed variables to be included in planned logistic regression models, Figure S1: Diagram of control arm and intervention arm, Figure S2: Formative research conducted to plan and develop intervention, Figure S3: Standard operating procedure (SOP) for pre- and post-intervention assessments, referred to as school surveys: contents, protocol, and workflow, Figure S4: Research portfolio give to school principals: contents and examples of documentation, Figure S5: Health education sessions for children: contents, including sample curriculum and lesson plan, and examples of PowerPoint slides, Film S1: “Hygiene Heroes”, locally-produced educational film promoting handwashing, Table S1: Intervention components: contents and distribution among schools in intervention arm, Table S2: Inclusion and exclusion criteria for schools and students, Table S3: Questions from health literacy questionnaire for primary school children, Table S4: Questions from health literacy questionnaire for secondary school children, Table S5: Questions from school WaSH and household questionnaire for children, Table S6: Compensation for control and intervention arms Table S7: Description of training workshops provided for research assistants, and Table S8: Strategies used to promote intervention adherence.
