# Supplementary material for: Protocol for a Trial Assessing the Impacts of School-Based WaSH Interventions on Children’s Health Literacy, Handwashing, and Nutrition Status in Low- and Middle-Income Countries
Source: Int J Environ Res Public Health. 2020 Dec 30;18(1):226. doi: 10.3390/ijerph18010226 (PMC7795080; doi:10.3390/ijerph18010226)
Supplement: Supplementary file 1 [file ijerph-18-00226-s001.pdf]

Supplementary Materials

Protocol for a Trial Assessing Impacts of School-Based WaSH Interventions on Children's Health Literacy, Handwashing, and Nutrition Status in Low- and Middle-Income Countries

Stephanie O. Sangalang, Shelley Anne J. Medina, Zheina J. Ottong, Allen Lemuel G. Lemence, Donrey Totanes, John Cedrick Valencia, Patricia Andrea A. Singson, Mikaela Olaguera, Nelissa O. Prado, Roezel Mari Z. Ocaña, Rovin James F. Canja, Alfem John T. Benolirao, Shyrill Mae F. Mariano, Jergil Gavieres, Clarisse P. Aquino, Edison C. Latag, Maria Vianca Jasmin C. Anglo, Christian Borgemeister, and Thomas Kistemann.

Table of Contents

Box S1. Policies relevant to WaSH in schools.
Box S2. Equation and proposed variables to be included in planned logistic regression models.
Figure S1. Diagram of control arm and intervention arm.
Figure S2. Formative research conducted to plan and develop intervention.
Figure S3. Standard operating procedure (SOP) for pre- and post-intervention assessments, referred to as school surveys: contents, protocol, and workflow.
Figure S4. Research portfolio give to school principals: contents and examples of documentation.
Figure S5. Health education sessions for children: contents, including sample curriculum and lesson plan, and examples of PowerPoint slides.
Film S1. "Hygiene Heroes", locally-produced educational film promoting handwashing.
Table S1. Intervention components: contents and distribution among schools in intervention arm.
Table S2. Inclusion and exclusion criteria for schools and students.
Table S3. Questions from health literacy questionnaire for primary school children.
Table S4. Questions from health literacy questionnaire for secondary school children.
Table S5. Questions from school WaSH and household questionnaire for children.
Table S6. Compensation for control and intervention arms.
Table S7. Description of training workshops provided for research assistants.
Table S8. Strategies used to promote intervention adherence.
References for Supplementary Materials

Box S1. Policies relevant to WaSH in schools.

SDG 6 [1]	DepEd Order Number 10 (2016) [2]
Ensure access to water and sanitation for all.<br><br>Targets:<br>6.1 By 2030, achieve universal and equitable access to safe and affordable drinking water for all.<br>6.2 By 2030, achieve access to adequate and equitable sanitation and hygiene for all and end open defecation, paying special attention to the needs of women and girls and those in vulnerable situations.<br>6.3 By 2030, improve water quality by reducing pollution, eliminating dumping and minimizing release of hazardous chemicals and materials, halving the proportion of untreated wastewater and substantially increasing recycling and safe reuse globally.	Policy and Guidelines for the Comprehensive Water, Sanitation and Hygiene in Schools (WinS) Program.<br><br>Targets:<br>1. Water: All schools shall have an organized system to make adequate and safe drinking water as well as clean water for handwashing, toilet use, menstrual hygiene management, and cleaning purposes available to all students during school hours;<br>2. Sanitation: All schools shall have adequate, clean, functional, safe, and accessible toilet facilities that meet the pupil-to-bowl ratio as stipulated in the Philippine Sanitation Code; maintain cleanliness and safety in and the immediate vicinity of school premises through school-based solid waste management, proper drainage, and the elimination of all possible breeding grounds for mosquitoes to prevent vector-borne diseases; and ensure safety in food handling and preparation;<br>3. Hygiene: All students in school shall perform supervised daily group handwashing with soap and tooth brushing with fluoride, while a system and support mechanisms for effective menstrual hygiene management shall be ensured in all schools;<br>4. Health Education: All teachers, heads of schools, facilities coordinators, and health personnel shall be oriented on the DepEd WinS program. Trained teachers can conduct Health Education in coordination with community leaders during Parent-Teacher Association (PTA) meetings. All pupils/students shall have a higher awareness of correct hygiene and sanitation practices and develop positive health behaviors;<br>5. Deworming: At least 85 percent of all students shall be dewormed semi-annually;<br>6. Capacity Building: All DepEd WinS program implementers shall undergo orientation on the program as needed.
Other SDGS[2]	
2. Zero Hunger.<br><br>2.1 By 2030, end hunger and ensure access by all people, in particular the poor and people in	3.4 By 2030, reduce by one third premature mortality from non-communicable diseases through prevention and treatment and promote mental health and well-being.

vulnerable situations, including infants, to safe, nutritious and sufficient food all year round.<br>2.2 By 2030, end all forms of malnutrition, including achieving, by 2025, the internationally agreed targets on stunting and wasting in children under 5 years of age, and address the nutritional needs of adolescent girls, pregnant and lactating women and older persons.<br><br>3. Ensure healthy lives and promote well-being for all at all ages.<br><br>3.3 By 2030, end the epidemics of AIDS, tuberculosis, malaria and neglected tropical diseases and combat hepatitis, water-borne diseases and other communicable diseases.	3.9 By 2030, substantially reduce the number of deaths and illnesses from hazardous chemicals and air, water and soil pollution and contamination.<br><br>4. Quality Education.<br><br>4.A Build and upgrade education facilities that are child, disability and gender sensitive and provide safe, nonviolent, inclusive and effective learning environments for all

Note: acquired immunodeficiency syndrome (AIDS); Department of Education Philippines = DepEd; parent-teacher association (PTA); Sustainable Development Goal (SDG); United Nations (UN); water, sanitation, and hygiene (WaSH); water, sanitation and hygiene in schools (WinS).
[1] Please see References for Supplementary Materials, 1.
[2] Please see References for Supplementary Materials, 2.

Box S2. Equation and proposed variables to be included in planned logistic regression models.

I. Equation

We will analyze the sample to identify and assess associations between outcomes and exposures (i.e. risk factors). We will use three logistic regression models: model A for poor hygiene literacy only, poor handwashing literacy only, and both poor hygiene and handwashing literacy; model B for malnutrition, i.e. stunting only, underweight only, and over-nutrition only; model C for poor health status only, extreme hunger only, and both poor health status and extreme hunger. We list our models' factors (variables) below. To measure associations between outcomes and exposures, we will use the following equation as the basis of our logistic regression models:

$$\log it(\pi_{abcdefg}) = \beta_1 San_b \times \beta_2 San_c + X c_{abc} + \Delta s_b + K s_d + F_f + H_g + \zeta_m$$

where

π_{abcde}	= dichotomous outcome for child *a* in school *b* in cluster *c* in survey *d* in matched group *e* from family *f* in home *g*
San_b	= whether school has any sanitation
San_c	= level of sanitation in school
C_{abc}	= vector of child characteristics
S_b	= vector of school characteristics
S_d	= vector of survey characteristics
F_f	= vector of family characteristics
H_g	= vector of home characteristics
ζ_m	= random intercept for matched group *m* that is assumed to be normally distributed with a mean of 0

II. Proposed variables

Model	Outcomes	Exposures
A	Poor hygiene literacy only, poor handwashing literacy only, and both poor hygiene literacy and poor handwashing literacy	Female, early-teenager [1], stunted only, over-nutrition only, is not satisfied with school restroom, school-level prevalence rates of: does not wash hands at school, avoids school restroom, does not use school restroom, lacks privacy, insufficient number of restrooms in school, long lines in school restroom, lack of hygiene lessons in school, number of school restrooms, number of school toilets, number of school handwashing basins, school restroom lacks water, is not clean, lack of policy to clean school restroom daily, maximum MOOE budget [2], no toilet at home, no running water at home, no electricity at home, family has no cellphone, second-hand smoke in home, parent cannot read
B	Stunting only, undernutrition only, and over-nutrition only	Female, pre-teenager [3], poor hygiene literacy, poor handwashing literacy, is not satisfied with school restroom, number of school toilets, number of school handwashing basins, school restroom lacks water, is not clean, lack of policy to clean school restroom daily, maximum MOOE budget [2], annual school enrollment < 2,000 students, no toilet at home, no running water at home, no electricity at home, family has no cellphone, second-hand smoke in home, parent cannot read, insufficient food at home, lack of food variety, family cannot afford food, family asks other people for money to buy

		food, family eats pre-cooked food or fast food more often than freshly cooked food
C	Poor health status only, extreme hunger only, both poor health status and extreme hunger	Female, pre-teenager [3], stunted only, poor hygiene literacy, poor handwashing literacy, is not satisfied with school restroom, number of school toilets, number of school handwashing basins, school restroom lacks water, is not clean, lack of policy to clean school restroom daily, maximum MOOE budget [2], annual school enrollment < 2,000 students, no toilet at home, no running water at home, no electricity at home, family has no cellphone, family has no watch or clock, second-hand smoke in home, parent cannot read, insufficient food at home, lack of food variety, family cannot afford food, family asks other people for money to buy food, family eats pre-cooked food or fast food more often than freshly cooked food

Note: maintenance and other operating expenses (MOOE); United States Dollar (USD).

[1] Early-teenager: 13 - 14 years old.

[2] Maximum MOOE budget in USD.

[3] Pre-teenager: < 13 years old.

Figure S1. Diagram of control arm and intervention arm.

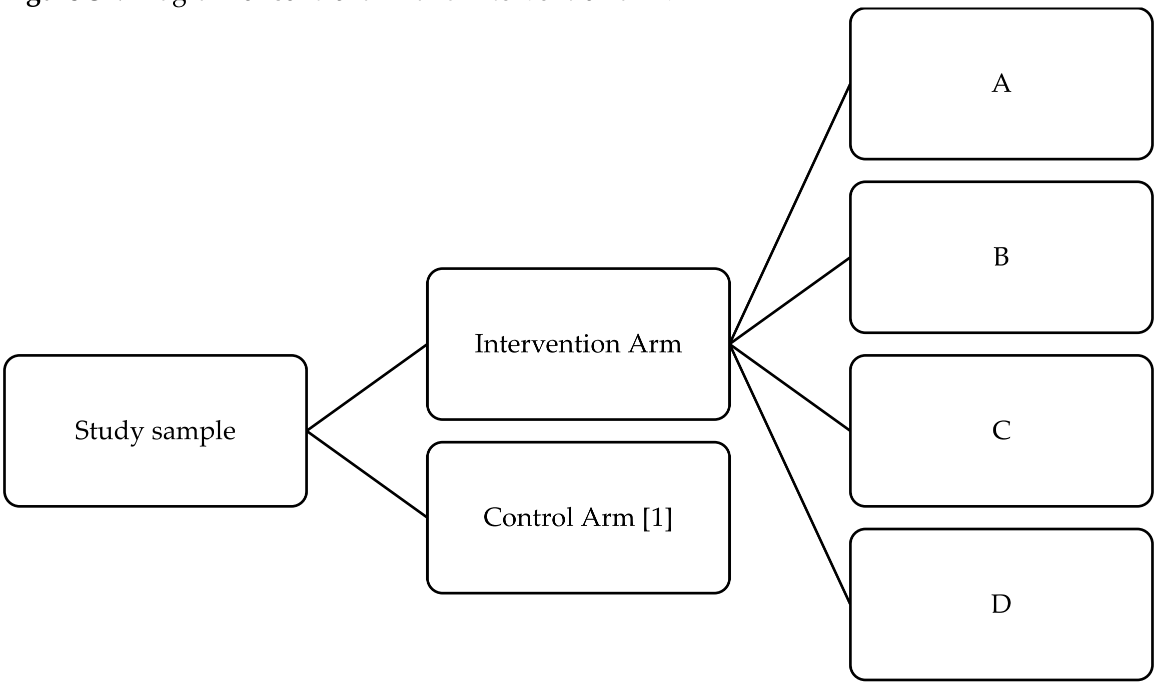

Intervention group	Intervention components received [2]
A	Policy recommendations, low-volume [3] health education, hygiene promotion workshop for teachers, poster contest, water purifier/dispenser for drinking water, and installation of toilets.
B	Policy recommendations, medium-volume [3] health education, hygiene promotion workshop for teachers, school restroom cleaning contest, hygiene supplies, and door locks for toilet cubicles.
C	Policy recommendations, high-volume [3] health education, hygiene promotion workshop for teachers, soap-making workshop, hygiene supplies, and repair of WaSH facilities.
D	Policy recommendations, medium-volume [3] health education, hygiene promotion workshop for teachers, poster contest, cleaning supplies for school janitors, and installation/construction of handwashing basins.

Note: water, sanitation, and hygiene (WaSH).
The intervention arm was divided into four groups based on the number of health education sessions (2-4) offered to study participants. Children did not know the schedule of intervention implementation in advance. It was not possible to completely blind them to the intervention as they directly received health education sessions and participated in classroom activities (e.g. contests). Many children were able to see the installation or repair of WaSH facilities, as these activities occurred on school grounds and sometimes during school-day hours. However, children were blinded to the provision of WaSH or hygiene supplies (e.g. liquid hand soap), which we gave directly to school principals or representatives without any children present.
[1] Control arm: received no intervention, rather the standard of care, which we defined as WaSH policy recommendations, hygiene promotion workshop for teachers, and two health education sessions for children.
[2] We provide a detailed description of intervention components in Table S1.
[3] Low-, medium-, and high-volume health education referred to children receiving 2, 3, and 4 one-hour sessions, respectively.

Figure S2. Formative research conducted to plan and develop intervention.

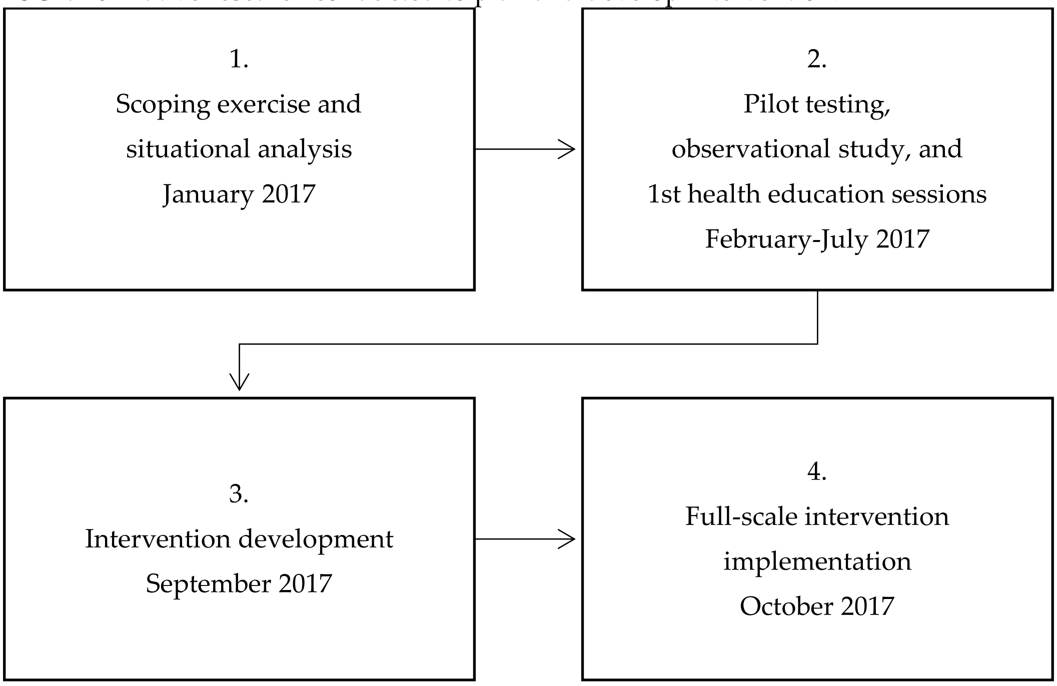

Note: water, sanitation, and hygiene (WaSH).

We conducted formative research to plan our strategy of developing, implementing, and evaluating a comprehensive school-based WaSH intervention package. We carried out formative research as follows: 1) Scoping exercise to identify potential study schools; conduct of situational analysis to describe gaps in school WaSH management and assess learning needs of children. 2) Pilot testing of survey instruments; conduct of observational study [1] to describe WaSH situation in 15 public schools and assess health, nutrition, and hygiene practices of children. Conducted 1st health education sessions in June-July 2017. (In one school from the control arm, we conducted the 1st health education session in October 2017 to accommodate the class's schedule). 3) Process of intervention development that was informed by findings from our observational study, inputs from local research assistants, and feedback from stakeholders. Engagement with the offices of city mayors, engineers, public health, and captains of "barangays" (Tagalog for "smallest units of local government").The latter were responsible for the day-to-day activities within neighborhoods wherein our study schools were located and where study participants lived. 4) Launch of full-scale intervention package in schools, which involved procurement and provision of WaSH equipment, supplies, and materials for facilities, e.g. toilets, urinals, and handwashing basins.

[1] Please see References for Supplementary Materials, 3.

Figure S3. Standard operating procedure (SOP) for pre- and post-intervention assessments, referred to as school surveys: contents, protocol, and workflow.

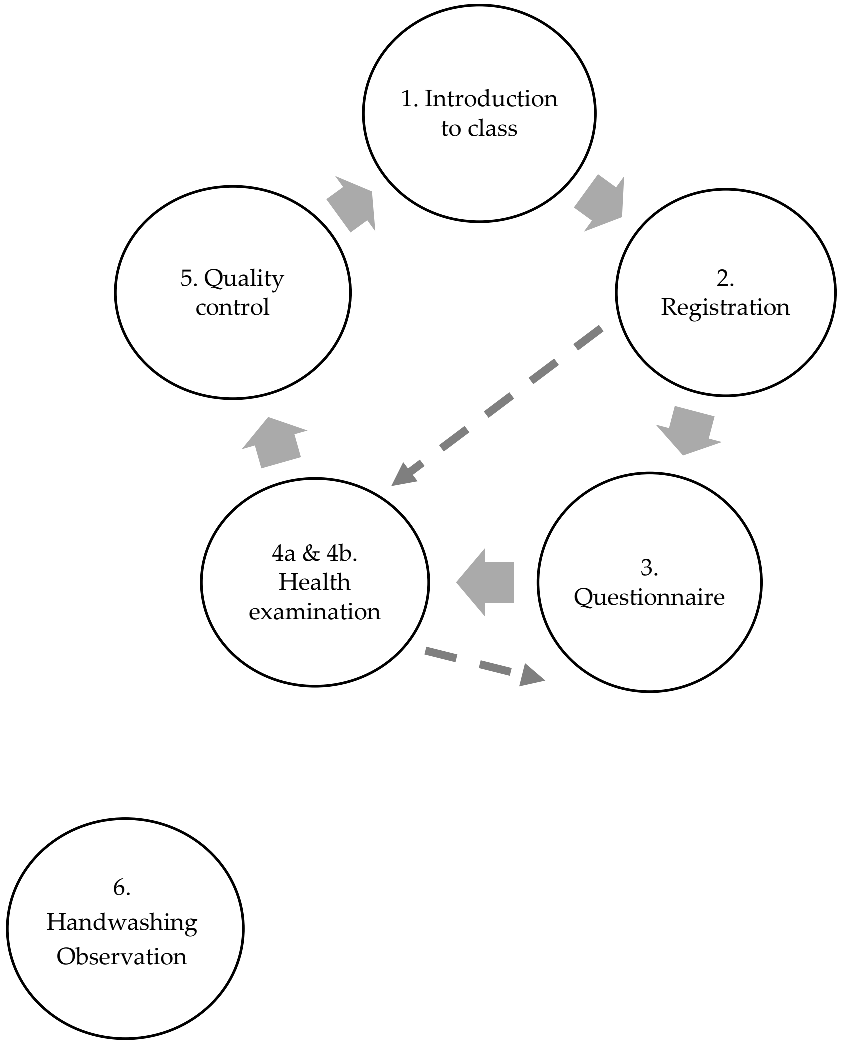

We conducted pre- and post-intervention assessments, referred to as school surveys, in June-July 2017 and February-March 2018. (We conducted the pre-intervention assessment at one school [from the control arm] in October 2017 to accommodate the class' schedule.) We assessed the same sample of children at baseline and at the 8-month follow-up.

Step	Station	Activities
1	Introduction	Upon arriving in the classroom, research assistants introduced themselves to the teacher and children. Research assistants explained, in Tagalog, the study's purpose, objectives, and methods. They explained that participation in the study was voluntary, anonymous, and confidential. They explained that data would not impact the children's school grades.

Step	Station	Activities
2	Registration	Research assistants asked children, one at a time, for his or her student identification (ID) number, date of birth, and telephone number. Then each child was assigned a unique study ID number. Data were inputted into a Master List (Excel file) stored on password-protected tablets or smartphones. Each child was given a set of 3 tickets containing his or her study ID number and instructed to wait for his or her turn to complete the health literacy questionnaire on an available tablet or smartphone. Due to the limited number of tablets and smartphones available, we dedicated at least one tablet to the height and weight station and one tablet to the urine station. All remaining tablets and smartphones were dedicated to the health literacy questionnaire station.
3	Questionnaire	Research assistants selected a group of 5-7 children, saying aloud the appropriate ID numbers, to begin the health literacy questionnaire. Children completed the health literacy questionnaire using an app installed on tablets and smartphones. Children answered the questionnaire independently and privately, away from the view of their classmates. Research assistants verified the completion of each child's questionnaire and collected from him or her the appropriately labelled ticket. Then the research assistant directed the child to proceed to the health examination station.
4	Health examination: height and weight	Research assistants measured children's standing height (to the nearest cm), without shoes, using a tape measure attached to the wall of the school building. We measured children's weight (to the nearest 0.1 kg), without shoes or any items inside their pockets, using a digital weighing scale (EKS Asia Ltd., Hong Kong, Special Administrative Region of the People's Republic of China). Research assistants inputted all data into the app.
	Health examination: urine	Research assistants gave children a plastic cup with their student ID number written on the outside of the cup. Research assistants asked children to take their cup to the restroom and fill the cup with their urine. Then research assistants received urine specimens and performed point-of-care urinalysis per protocol. According to the manufacturer's instructions, we dipped for at least 5-6 seconds one urine test strip (Insight Urinalysis Reagent Strips, Acon Laboratories Inc., San Diego, California, U.S.A.) into one urine specimen. After waiting at least 2-3 minutes, we interpreted the urinalysis results by comparing the color changes displayed on the urine test strip to the manufacturer-provided urinalysis interpretation guide. Urinalysis test results were interpreted by one research assistant and inputted into the app by another research assistant. We disposed of urine specimens, cups, and urine test strips according to protocol, immediately after

		completing our survey. Alternatively, at the beginning of the school survey, research assistants would call a group of 5-7 students to participate in the health examination before completing the health literacy questionnaire. Afterward, the students would be directed to complete the health literacy questionnaire.
5	Quality control	As a quality control measure, at the end of the school survey, but prior to leaving the school campus, we counted the number of responses saved in the tablets and smartphones. We verified that this number matched the total number of tickets collected from all stations (questionnaire, height and weight, and urine stations). This helped us to identify missing data if a child did not provide all the required data. If needed, then we would approach the child and ask him or her to provide the missing data.
6	Handwashing observation	We conducted unannounced handwashing observations directly after a school survey or on days where no school survey was conducted. We observed children who may or may not have participated in our school survey as described above (steps 1-4). To conduct the handwashing observations, we positioned ourselves inside or at the doorway of school restrooms to visualize whether children washed their hands after using the toilet or urinal, and if they used the correct handwashing technique and for the correct duration of time (<= 20 seconds). We calculated school-level handwashing prevalence rates by taking the mean score of all observations performed at the same school.

Note: less than or equal to (<=); centimeter (cm); identification (ID); kilogram (kg); limited company (Ltd.); United States of America (U.S.A.).

Figure S4. Research portfolio give to school principals: contents and examples of documentation.

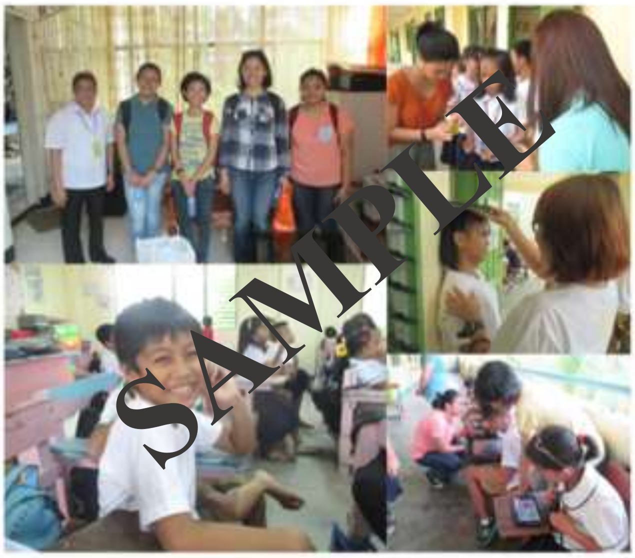

Research Portfolio for [redacted]

**Project Name**	Impact of inadequate water, sanitation, and hygiene, and food insecurity on children in public schools in Metro Manila.
**Research Institutions**	Institute of Environmental Science and Meteorology, University of the Philippines, Diliman; Center for Development Research, University of Bonn, Germany.
**Research Dates**	February 2017 – April 2018

Contents of Research Portfolio

I. Acknowledgements
II. Project partners
III. Summary
IV. Timeline
V. Research questions
VI. Research objectives
VII. Research methods
VIII. Research findings
IX. Recommendations/action plan
X. Appendix
  i. Memos
  ii. Photos
  iii. Data summaries

Data collection

Electronic tablets loaded with an app were used to collect data from:

a. questionnaires and interviews from stakeholders (principals, students)
b. students' health exams (height, weight, urinalysis)
c. inspections of school and home restrooms.
d. The water quality testing will be conducted on April 2018.

Research Findings

Summary of Main Findings

Availability
- L[illegible]esources
- No [illegible] water, soap, tissue paper, or towels

Access
- Lack of wheelchair ramps

Quality
- Many equipment/facilities are not fully operational or well maintained

Quantity
- Too many students, too few toilets/urinals

SAMPLE

Study Population

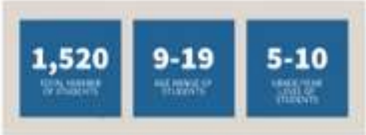

In the first phase of the study, there was a total number of 1, [illegible] respondents with the age range of 9 – 19 and with the grade level [illegible] – 10.

Among these respondents, **93%** use[illegible]school [illegible]estrooms, **84%** do not avoid using the restrooms but **only 48% are [illegible]** [illegible] the schools' restrooms.

Adequacy of WASH [illegible] school

The availability of th[illegible] comfort rooms. The quality of the comfort room:

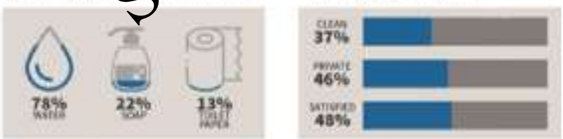

The accessibility of the comfort room:

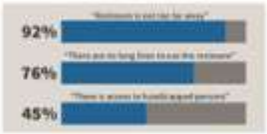

SAMPLE

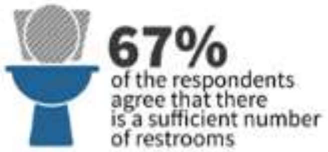

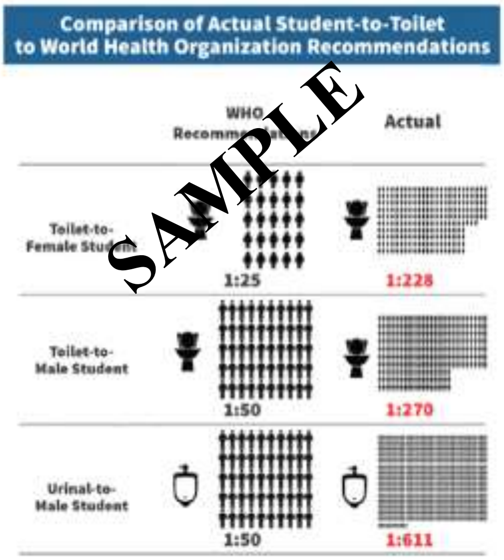

SAMPLE

Includes preliminary results. Please do not cite, disseminate, publish, or quote.

Key Findings for [redacted]
(More data to follow)

Total Enrollment (6):	1989
Males:	1057
Females:	932
Grade/No. of students in Phase 1:	5/90
No. of students' households:	5
Grade/No. of students in Phase 2:	6/82
Grade/No. of students in Phase 3: (to follow).	

INDICATOR	RESULT n= 90
toilet-to-female student ratio [7]	1:310
toilet-to-male student ratio	0:1057
urinal-to-male student ratio	0:1057
% of students who use school res[illegible]	97%
% of students who are satis[illegible] s[illegible]ol restroom	67%
% of students who said [illegible]ool r[illegible]ooms are clean	38%
% of students wh[illegible] s[illegible]estrooms are accessible	46%
% of students wh[illegible] school restrooms provide enough privacy	64%
% of students who wash hands at school	88%
% of students who wash their hands with soap	22%
% of students who are satisfied with school hand washing area	38%
% of students who said school hand washing area is clean	30%
% of students who said water is available	42%
% of students who said soap is available	16%
% of students who considered themselves to be "healthy"	76%
% of students who have not been absent from school due to health problems	49%

[6] Source: DepEd, SY 2015-2016 Public Schools Enrollment.
[7] Toilet-to-females: 3:332; toilet-to-males: 0:1,057; urinals-to-males: 0:1,057.

SAMPLE

Figure S5. Health education sessions for children: contents, including sample curriculum and lesson plan, and examples of PowerPoint slides.

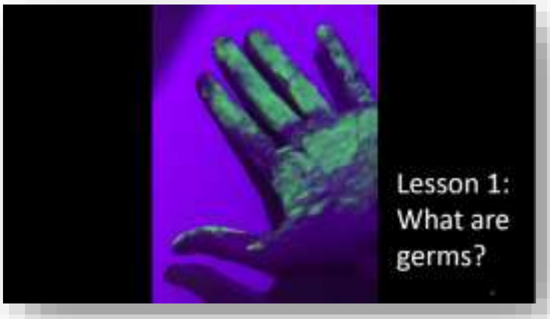

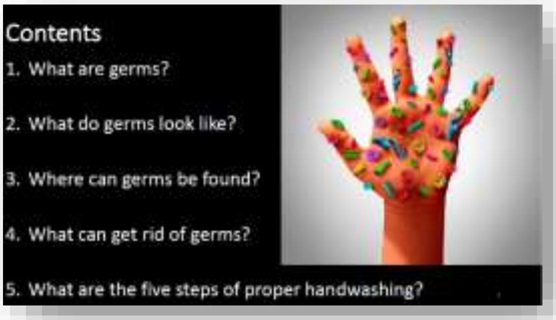

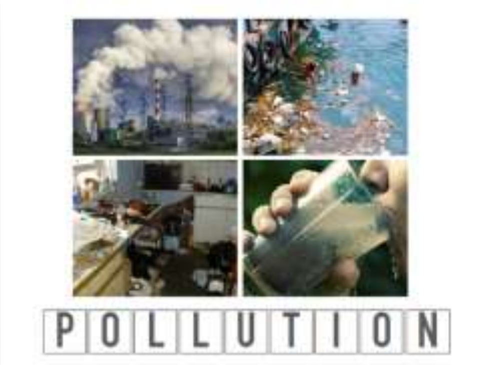

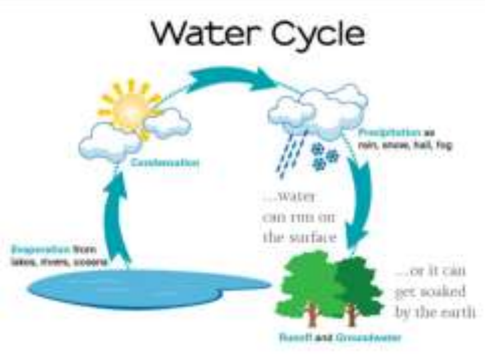

Contents of Health Education Sessions

I. Overview of health education curriculum
- A. Lesson 1: What are germs?
- B. Lesson 2: What is environmental health?
- C. Lesson 3: Climate safety
- D. Lesson 4: Health promotion

II. Sample lesson plan for Lesson 2: What is environmental health?
- A. Introduction
  - 1. Describe different environments. (show photos and ask students to name places)
    - a. Colorado Rocky Mountains
    - b. Paris nightlife
    - c. Golden Gate Bridge
    - d. Egyptian pyramids
    - e. Beauty of the Philippines
      - i. Boracay beaches
      - ii. Chocolate Hills, Bohol
      - iii. Green hills of Batanes
  - 2. List 4 things that we all need to live: air, water, food, shelter.
  - 3. What impacts do these things have on our health?
    - a. Air: provides oxygen → but can cause breathing problems/allergies/asthma if…
    - b. Water: provides hydration → but can cause diseases/diarrhea if…
    - c. Food: provides nutrients → but can cause diseases/diarrhea if…
    - d. Shelter: provides safety/security → but can cause diseases/diarrhea if…
- B. Case study #1: Jane breathes in air pollution

1. Scenario: Jane is your schoolmate but from a different class section. She lives about 10 minutes away from you. She walks or takes the jeep to school. Her hobby is to go to the mall with her friends. She has had asthma (“hika” in Tagalog) since she was 6 or 7 years old. But it got worse 2 weeks ago, when she decided to stop using her face mask while riding the jeep. Now she is coughing at night, breathing loudly during exercise, and sometimes cannot catch her breath.

2. What causes air pollution? Man-made activities
   - a. burning things
   - b. spraying chemicals
   - c. cooking with coal
   - d. using equipment/vehicles that produce emissions

3. What are the health effects? Lung problems
   - a. irritation/allergy
   - b. asthma
   - c. infections like pneumonia

4. Discussion questions
   - a. Why do you think Jane stopped wearing her face mask? Was it a good idea or not? Why?
   - b. What are the main causes of Jane’s breathing problems? What can she do to cure her breathing problems? What can she do to prevent them from happening again in the future?
   - c. If you could eliminate one of the four man-made causes of air pollution, then which one would you choose. Why?

C. Case study #2: Justin drinks “dirty” water

1. Scenario: Justin is your schoolmate but in the grade below you. He lives near the river and his hobby is playing sports (basketball, jogging). Last week he decided to stop drinking bottled water because it is expensive and he thinks he is “immune” to the germs in the tap water. Now he drinks water directly from the tap. Three days ago he started to have loose bowel movements (known as “LBM”, another term for “diarrhea”). Today he missed school because he got dehydrated.

2. What causes water contamination?
   - a. Natural causes: animal feces or corpses
   - b. Man-made causes: urine, feces, food, garbage, chemicals

3. What are the health effects? diarrhea, fever, infection

4. Discussion questions
   - a. If Justin lives near the river, then what could be some causes of water contamination?
   - b. Why did he think he was “immune” to the germs in the water? Was he right or wrong? How do you know?
   - c. Why did he get LBM? Why is LBM dangerous?
   - d. If bottled drinking water is too expensive, then what advice would you give Justin to prevent this problem in the future?
   - e. Do you think having clean drinking water is a right? Should it be free or should we have to pay for it?

D. Case study #3: Amy eats “dirty” food

1. Scenario: Amy is your “ate’s” (Tagalog for “older sister”) best friend and she lives just next door to you. Her hobby is watching Korean telenovelas. Yesterday after school, Amy ate fish balls cooked by a vendor who was new to your town. One hour later, she started to have a painful stomachache. At night, she was vomiting and having a high fever.

2. What causes food contamination?

a. Natural causes: animal feces or corpses

b. Man-made causes: urine, feces, food, garbage, chemicals

3. What are the health effects? diarrhea, vomiting, fever, infection

4. Discussion questions

a. Do you think it is safe to eat street food? Why/why not?

b. Why is it important to know (the vendor) who is cooking your food?

c. Do you or your family cook fresh food more often than buying already cooked food?

d. What should Amy do now that she is so sick? What can she do to prevent this in the future?

E. Case study #4: Bryan lives in a "dirty" house

1. Scenario: Bryan is the one of the most popular students at your school because he is very funny. He lives behind the school but is often home alone because his parents work a lot. Last weekend you visited his house and noticed that there were dirty dishes in the kitchen sink, and ants and flies near the garbage. There were also used diapers, from Bryan's baby sister, on the floor. The C.R. (known as "comfort room", another term for "restroom") smelled very badly--like old urine.

2. What causes a house to become dirty? man-made causes like lack of cleaning or improper sanitation/hygiene

3. What are the health effects? allergy, diarrhea, spread of infection

4. Discussion questions

a. What could be the main cause of the problems in Bryan's house?

b. Could you give Bryan advice about how to clean his house?

c. What improvements could you make at your own house?

d. What do you think is more important: cleanliness of oneself or cleanliness one's environment? Why?

F. Activity: work in small groups

1. Choose a topic: air, water, food, or shelter

2. Create a case study based on your school/community

3. List 2 questions you can ask the class

4. All groups share their case studies

G. Quiz game (if time permits)

III. References [1]

Note: comfort room (i.e. restroom) (C.R.); loose bowel movement (i.e. diarrhea) (LBM).

[1] Please see References for Supplementary Materials, 4-7.

Film S1. "Hygiene Heroes", locally-produced educational film promoting handwashing.

(File attached.)

In response to the learning needs related to handwashing that we identified during our previous observational study [1], we created an educational film to promote handwashing. Research assistants (all of whom are Filipino college students or recent graduates) acted in the film, speaking in Tagalog. All scenes were filmed on the University of the Philippines Diliman campus. The film was about a girl who experienced nausea and vomiting after not properly washing her hands before eating her lunch. She had a dream about germs living inside someone's body and the germs were causing an internal war. Two superheroes, named Water and Soap, teamed up to defeat the germs in battle. We showed the film to 1-3 class sections per school (about 50 students per class section) in 15 schools, and we estimate that over 1,100 children viewed the film.

---

[1] Please see References for Supplementary Materials, 3.

Table S1. Intervention components: contents and distribution among schools in intervention arm.

Components of WaSH intervention package			
WaSH policies	Health education	Hygiene supplies	WaSH Facilities' improvements

Group	Intervention components (by number)															Number of schools allocated to intervention group (A-D)
	1	2	3	4	5	6	7	8	9	10	11	12	13	14	15	
Intervention group A	X	X			X		X				X				X	2
Intervention group B	X		X		X			X		X				X		3
Intervention group C	X			X	X	X				X		X				4
Intervention group D	X		X		X		X		X				X			4
Total number of schools																13 [1]

Intervention component number	Intervention type	Description of intervention activities	Rationale for intervention
1	Policy	Policy recommendations	Lack of policy or weak enforcement
2	Education	Low-volume health education: 2 one-hour classroom sessions	Lack of knowledge or ineffective hygiene behavior
3	Education	Medium-volume health education: 3 one-hour classroom sessions	Lack of knowledge or ineffective hygiene behavior
4	Education	High-volume health education: 4 one-hour classroom sessions	Lack of knowledge or ineffective hygiene behavior
5	Education	Hygiene promotion workshop for teachers: 1 one-hour classroom session	Lack of knowledge
6	Education, hands-on activity	Soap-making workshop for parents: 1 one-hour lesson	Lack of soap, lack of knowledge, or ineffective hygiene behavior
7	Education, hands-on activity	Poster contest	Lack of knowledge
8	Education, hands-on activity	School restroom cleaning contest	Lack of cleanliness or ineffective hygiene behavior

9	Supplies [2]	Package of cleaning supplies for school janitors	Lack of supplies
10	Supplies [2]	Package of hygiene supplies for children	Lack of supplies
11	Supplies [2]	Water purifier/dispenser for drinking water	Lack of drinking water
12	Facilities	Repair of WaSH facilities	Broken WaSH facilities
13	Facilities	Installation/construction of handwashing basins	Lack of handwashing basin or running water for handwashing
14	Facilities	Installation of door locks for toilet cubicles	Lack of privacy in toilet cubicles
15	Facilities	Installation of toilets	Insufficient number of toilets

Note: water, sanitation, and hygiene (WaSH).
In Microsoft© Excel we listed the intervention components (numbers 1-15) and estimated each component's required resources (time, budget, and personnel). We then categorized the 15 intervention components into four groups (A, B, C, and D). We determined how many schools would be allocated to each intervention group based on our available resources for time, budget, and personnel. Each intervention group included the standard of care (also given to the control arm), as described below, plus low-, medium-, or high-volume[2] health education sessions and different combinations of hands-on activities, supplies, and hardware.
[1] This does not include the two schools from the control arm. The control arm received no intervention, rather the standard of care, which we defined as WaSH-related policy recommendations, a hygiene promotion workshop for teachers, and two health education sessions for children (these overlap with intervention component numbers 1, 2, and 5).
[2] See Table S6 for description of supplies provided.

Table S2. Inclusion and exclusion criteria for schools and students.

Inclusion criteria	Exclusion criteria
Schools	Schools
1. Classrooms are accessible during school-day hours.	1. Classrooms are not accessible during school-day hours.
2. WaSH facilities, e.g. toilets, urinals, and handwashing basins, are available on school grounds.	2. No WaSH facilities, e.g. toilets, urinals, or handwashing basins, available on school grounds.
Students	Students
1. Enrolled in school.	1. Not enrolled in school.
2. Primary school: grade 5 or 6.	2. Primary school: not in grade 5 or 6.
3. Secondary school: grade 7, 9, or 10.	3. Secondary school: not in grade 7, 9, or 10.
4. Able to comprehend and complete questionnaire, using tablet or smartphone, independently or with minimal assistance.	4. Unable to comprehend or complete questionnaire, using tablet or smartphone, independently or with minimal assistance.
5. Able to be measured for height and weight.	5. Unable to be measured for height and weight.
6. Able to provide urine specimen.	6. Unable to provide urine specimen.

Table S3. Questions from health literacy questionnaire for primary school children.

Number	Question
1	What is the date today?
2	When were you born?
3	How old are you?
4	What grade are you in?
5	Are you male or female?
6	What is your full address?
7	What is the telephone number of your parent/guardian?
8	What is the name of your school?
9	Did you attend this school during the last school year?
10	What are germs? They are small things that can: (choose only one answer).
11	Where can germs be found? (Read carefully the answer options and choose only one answer.)
12	What can get rid of germs? (Read carefully the answer options and choose only one answer.)
13	How can germs be transmitted from one person to another person? (Read carefully the answer options and choose only one answer.)
14	True or false: If I have germs, then I can have vomiting or diarrhea.
15	True or false: If I catch germs, then I can get an infection or sore throat.
16	True or false: Washing hands with soap and water can prevent the spread of germs.
17	When should I wash my hands with soap and water? (Read carefully the answer options and choose only one answer.)
18	How long should I wash my hands with soap and water to get rid of germs? (Choose only one answer.)
19	True or false: Washing fruits and vegetables before cooking or eating them is one way to prevent the spread of germs.
20	True or false: If the restroom is not clean, then it may contain germs which can cause illness.

Table S4. Questions from health literacy questionnaire for secondary school children.

Number	Question
1	What is the date today?
2	When were you born?
3	How old are you?
4	What grade are you in?
5	Are you a male or female?
6	What is your full address?
7	What is the telephone number of your parent/guardian?
8	Where do you go to school?
9	Did you attend this school during the last school year?
10	What are germs? They are small things that can: (choose only one answer).
11	Where can germs be found? (Read carefully the answer options and choose only one answer.)
12	What can get rid of germs? (Read carefully the answer options and choose only one answer.)
13	How can germs be transmitted from one person to another person? (Read carefully the answer options and choose only one answer.)
14	If I have germs, then I can have: (this/these symptom/symptoms).
15	What can prevent the spread of germs?
16	When should I wash my hands with soap and water? (Read carefully the answer options and choose one answer.)
17	How long should I wash my hands with soap and water to get rid of germs? (Choose only one answer.)
18	Why should we wash fruits and vegetables before cooking or eating them?
19	What could happen if the school restroom is not properly cleaned?
20	Why are there many germs inside the school restroom?

Table S5. Questions from school WaSH and household questionnaire for children.

Number	Question
1	Date
2	What is your study ID number?
3	What is your date of birth?
4	Are you male or female?
5	What is the telephone number of your parent/guardian?
6	How old are you?
7	In your opinion, are you healthy?
8	What is the name of your school?
9	Are you satisfied with the condition of the school restrooms?
10	Are the school restrooms clean?
11	Are you satisfied with the handwashing area at school?
12	Are the handwashing areas at school clean?
13	Is there a restroom in your home?
14	Do you share your restroom with another family?
15	Do you need to go outside your house to use the restroom?
16	Does your home have a toilet?
17	Does your home have a handwashing basin to wash hands?
18	Does your home have a faucet with running water?
19	Where does your family get its drinking water? (You may choose > 1 answer.)
20	Does your home have electricity?
21	What type of material is your home's floor made of? (Choose only 1 answer.)
22	Does your family have a refrigerator?
23	Does your family have its own cellphone?
24	Does your family have its own computer?
25	Does your family have its own watch/clock?
26	What types of transportation vehicles does your family have? (You may choose > 1 answer.)
27	Which mode of transportation do you most often use to go to school? (Choose only 1 answer.)
28	Does your family have its own electric fan?
29	How many adults (>= 18 years old) live in your home?
30	How many children (<= 17 years old) live in your home?
31	Is there anyone who smokes in your family?
32	Is there anyone who smokes inside the house?
33	How many adults (>= 18 years old) from your home work?
34	How many children (<= 17 years old) from your home work?
35	What is the highest level of education attained by your parent/guardian? (Choose only 1 answer.)
36	Do you have a parent/guardian who cannot read?
37	Do your parents/guardians have a physical or psychological disability that prevents them from working?
38	Do you often have enough food for everyone to eat at home?
39	Do you often have a variety of food (for example: fruits, vegetables, or meat like pork, chicken or fish) to eat at home?
40	Can you family often afford to buy food?

41	Does your family often NOT ask other people for money to buy food?
42	Does your family often cook its own food rather than buy pre-cooked food from a canteen or fast food?
43	Do you often go to sleep without feeling hunger in your stomach?
44	Do you have any questions or comments? Please write your questions or comments.

Note: identification number (ID); less than or equal to (<=); more than (>); more than or equal to (>=).

Table S6. Compensation for control and intervention arms.

Control arm [1] compensation package	Intervention arm compensation package	
Viewing of "Hygiene Heroes" video [2]; hand hygiene gel [3]	Group A	Control arm compensation package plus poster contest prizes [4], educational workbook [5]; ceramic water filter for drinking water (ID 8) [6], and repair and installation of toilets (ID 14).
	Group B	Control arm compensation package plus school restroom cleaning contest prizes [7], hygiene supplies [8], door locks for toilet cubicles; educational workbooks (ID 3) [5], and handwashing basins (ID 4) [9].
	Group C	Control arm compensation package plus hygiene supplies [8], school supplies (ID 2) [10], educational workbooks (ID 2,5,9) [5], soap-making materials (ID 2,5,11) [11], handwashing basins (ID 5,11) [9], and repair/installation of WaSH facilities (ID 2,5) [12].
	Group D	Control arm compensation package plus poster contest prizes [4], restroom cleaning supplies [13], educational workbook (ID 6,10,12) [5], school supplies (ID 6,10,12) [10], handwashing basins (ID 7,12) [9], gravity driven membrane filtered drinking water dispenser (ID 6,7) [14]; and menstruation hygiene supplies [15], shelving, garbage cans (ID 10).

Note: Department of Science and Technology Philippines (DOST); gravity-driven membrane (GDM); Gwangju Institute of Science and Technology (GIST); identification (number) (ID); International Environmental Research Institute (IERI); water, sanitation, and hygiene (WaSH); World Wildlife Fund (WWF).

The ID numbers refer to schools that participated in the WaSH in Manila Schools project.

[1] Rationale for compensation of control arm: 1) to meet appropriate ethical considerations regarding the handling of control participants; 2) to recompense control participants for the burden of partaking in our study, while reducing losses to follow-up; and 3) to offset some of the economic advantage received by intervention participants by providing the standard of care (i.e. WaSH-related policy recommendations, hygiene promotion workshop for teachers, and 2 health education sessions for children). Additionally, after the project was completed, we donated school supplies (as described below) to one of the two schools, having children with the most need, in the control arm and hand towels to both schools in the control arm.

[2] ″Hygiene Heroes": educational video, promoting handwashing, filmed by and featuring Filipino research assistants speaking in Tagalog.

[3] Hand hygiene gel: made from raw materials and bottled by research assistants.

[4] Poster contest prizes: certificate, medal, and school supplies.

[5] Educational workbook: containing information about environmental conservation; donated by the WWF Philippines.

[6] Ceramic filter for drinking water: locally developed technology (by the University of Northern Philippines in Vigan City, Ilocos Sur) that used local materials (red clay). Able to filter tap or deep well water, at the rate of 2 liters per hour, for up to one year. Units donated by the DOST Philippines.

[7] School restroom cleaning contest prizes: snack foods (e.g. cupcakes, ice cream) and drinks.

[8] Hygiene supplies: liquid hand soap, soap dispenser, toilet paper, and paper towels.

[9] Handwashing basins: construction of freestanding or wall-mounted unit with single or multiple (2-6) handwashing basins connected to running water.

[10] School supplies: pen, pencil, notebook, coloring pencils, and reusable tote bag.

[11] Soap-making materials: gloves, plastic soap molds, and raw materials (oil, lye, fragrance, and pink color dye).

[12] Repair/installation of WaSH facilities: using donated or brand new toilets, urinals, and handwashing basins.

[13] Restroom cleaning supplies: all-purpose cleaner, bucket, mop, broom, dustbin, sponge, and wash cloths.

[14] GDM-filtered drinking water dispenser: donated by the IERI at the GIST, South Korea.

[15] Menstruation hygiene supplies: sanitary napkins ("pads") and panty liners.

Table S7. Description of training workshops provided for research assistants.

Training workshop	Instructor(s)	Contents	Instruction methods	Duration
1. For all newly hired research assistants	Research supervisor with assistance from experienced [1] research assistants	1. Introduction, icebreaker, and work documents<br>2. Problem background<br>3. Study purpose and objectives<br>4. Review of previous study phases<br>5. Research methods and materials used in school survey<br>6. Effective communication (including role playing)<br>7. Teamwork<br>8. Classroom management<br>9. Field work 101<br>10. Research ethics<br>11. Work contracts and time sheets<br>12. Next steps	1. Interactive lecture using PowerPoint<br>2. Hands-on demonstration<br>3. Role playing	One full day (8 hours)
2. Making soap and hand hygiene gel	Initially a research assistant (chemist); then, the research supervisor	1. Preparation of work space<br>2. Safe handling of raw materials<br>3. Step-by-step procedure for making soap and hand hygiene gel<br>4. Storage and clean up	1. Hands-on demonstration	One hour
3. Data processing	Research supervisor	1. Overview of data management<br>2. Safe handling and storage of data<br>3. Data security<br>4. Review of Excel basics	1. Hands-on demonstration, with research assistants working directly on their own laptops	Full day (8 hours)

		5. Data processing: cleaning and coding<br>6. Documentation of discrepancies<br>7. Preparation of research summary<br>8. Creating and formatting research portfolios		
4. Water quality testing	Research supervisor, with input from technical experts [2]	1. Field visit to dams and water treatment facilities<br>2. Step-by-step procedure for collecting, labeling, and transporting water samples<br>3. Step-by-step procedure for conducting select water quality tests in laboratory<br>4. Step-by-step procedure for assessing water for coliform and *E.coli* contamination	1. Hands-on demonstration	3 full days (8 hours each)

Note: *Escherichia coli* (*E.coli*); Gwangju Institute of Science and Technology (GIST); Institute of Environmental Science and Meteorology (IESM); University of the Philippines (U.P.); water, sanitation, and hygiene (WaSH).
[1] Experienced research assistants were those who had previously worked on the WaSH in Manila Schools project. We hired different teams of research assistants, with 5-8 people per team, for different phases of the project. This made it possible for experienced research assistants to help train newly hired research assistants during subsequent project phases, while maintaining continuity in the work atmosphere. The research supervisor directly managed the research team, in person, from the beginning to the end of the project.
[2] Technical experts, from the IESM, U.P. Diliman, the GIST, and Manila Water Company, Inc., voluntarily offered guidance related to water quality testing.

Table S8. Strategies used to promote intervention adherence.

Communication method	Person(s) contacted	Activities	Frequency
Phone call or SMS text	School principal and teachers	Confirm schedules for intervention activities	1-2 times per month, with one call or SMS text 1-2 days before scheduled activity
Face-to-face meeting in office	School principal	Discuss detailed implementation of intervention and conclusion of intervention	2 times (once at beginning and once at end of project)
Face-to-face meeting on school campus	School personnel responsible for WaSH facilities	Discuss repair or installation of toilets, urinals, or handwashing basins	2 times (once at beginning and once at end of project), with one call or SMS text 1-2 days before scheduled repair or installation
Face-to-face meeting in classroom	School children [1]	Conduct of health education sessions, poster and school restroom cleaning contests, and songwriting workshop	Various: 2-8 meetings, depending on type of intervention received
Face-to-face meeting on school campus	Other school personnel [2]: secretaries, nurse, canteen workers, security guards	Monitor progress of WaSH facilities repairs, installations, and new construction	Various: 1-4 meetings, depending on type of intervention received

Note: short message service (SMS); water, sanitation, and hygiene (WaSH).

[1] Communicating with school children: We made it a point to spend quality time with them, to pay attention to their feedback, validate their concerns, and encourage them.

[2] We made an effort to be respectful of and friendly with all school personnel because we wanted to be viewed as people who wanted to help their students, rather than as inspectors or auditors. This was important for fostering a collaborative, trusting, long-term work relationship.

References for Supplementary Materials

1. UN. *Transforming our world: The 2030 Agenda for Sustainable Development*; United Nations: New York, USA, 2015.
2. DepEd. *DepEd Order No. 10, s. 26. Policy and Guidelines for the Comprehensive Water, Sanitation, and Hygiene in Schools (WinS) Program*; Department of Education: Pasig City, Philippines, 2016.
3. Sangalang, S.; Prado, N.; Lemence, A.; Cayetano, M.; Lu, J.; Valencia, J.; Kistemann, T.; Borgemeister, C. Diarrhea, Helminth Infection, Dehydration, and Malnutrition Associated with Water, Sanitation, and Hygiene Facilities and Poor Handwashing in Schools in Metro Manila, Philippines: A Cross-Sectional Study. *Am J Trop Med Hyg* (unpublished; manuscript under review).
4. DepEd. *K to 12 Curriculum Guide: Health. Grade 1 to Grade 10*; Department of Education: Pasig City, Philippines, 2012.
5. EPA. *Recipes for Healthy Kids and a Healthy Environment Kids Building a Safer and Healthier Community*; United States Environmental Protection Agency: Washington, D.C., USA, 2013.
6. U.S. National Library of Medicine. Available online: https://kidsenvirohealth.nlm.nih.gov/subtopic/004/water-pollution/009/water-cycle/ 2/3 (accessed on 8 October 2017). (as of 09/24/2020, webpage has moved to unknown location)
7. U.S. National Library of Medicine. Available online: https://kidsenvirohealth.nlm.nih.gov/subtopic/004/water-pollution/008/waterborne-diseases-&-illnesses/ 2/2 (accessed on 8 October 2017). (as of 09/24/2020, webpage has moved to unknown location)
